# Supplementary material for: Development and validation of the MIPPE: A novel dyadic assessment tool for early parent-child interactions in clinical practice
Source: PLoS One. 2026 Apr 24;21(4):e0347521. doi: 10.1371/journal.pone.0347521 (PMC13108784; doi:10.1371/journal.pone.0347521)
Supplement: S1 File — Complete version of the Measure of Early Parent-Child Interactions (MIPPE), including all 9 items and scoring instructions. (PDF) [file pone.0347521.s001.pdf]

Si vous utilisez l'échelle, merci de référencer la revue *PLOS ONE*. Vous pouvez nous faire des retours dans un but d'amélioration via : [secretariat.pedopsychiatrie-brabois@cpn-laxou.com].

When using this scale, please reference the PLOS ONE journal. Feedback for improvement purposes can be sent to: [secretariat.pedopsychiatrie-brabois@cpn-laxou.com].

Annexe 1

MIPPE

Mesure des Interactions Précoces Parents-Enfants

La cotation doit faire suite à un moment d'interaction entre le parent et son enfant (partenaires), d'au minimum 10 minutes : jeu, bain, change, activité, repas (à l'exception du biberon ou de l'allaitement).

0 correspond à une interaction de très mauvaise qualité et 3 à une interaction de très bonne qualité.

Les signes (+), (+/-), (-/+), (-) **qui se trouvent à la fin des items sont des repères visuels pour nuancer la cotation.**

Si hésitation entre 2 items, coter l'item le plus négatif.

La cotation doit prendre en compte le niveau de développement de l'enfant et la situation. Par exemple, lorsque l'enfant teste les limites, il est attendu de la part du parent qu'il utilise un ton plus ferme, que sa position du corps se modifie, etc.

Date	
Nombre de consultations au moment de l'évaluation	

1. Qualité des interactions visuelles

- [ ] Les échanges visuels sont toujours ajustés, fréquents, maintenus, spontanés et permettent une interaction de qualité entre les deux partenaires. Les regards sont adressés, dans une dynamique d'échange qui témoigne d'une attention réciproque. (+)

- [ ] Les échanges visuels soutiennent souvent une interaction de qualité. L'un des deux partenaires peut être plus actif dans la recherche de ces échanges. L'interaction visuelle ne témoigne pas toujours d'une attention réciproque de l'un envers l'autre, elle peut être fluctuante. (+/-)

- [ ] Les échanges visuels permettent parfois une interaction de qualité et sont majoritairement inadaptés (brefs, mécaniques, sur un versant négatif). La qualité des échanges visuels est pauvre. Le contact est vague et fugace. (-/+)

- [ ] Aucune interaction visuelle durant l'interaction : refus ou évitement du contact visuel. (-)

2. Qualité des interactions vocales

- [ ] Les échanges vocaux sont toujours ajustés, fréquents, maintenus, modulés, positifs et spontanés des deux partenaires. Ils permettent une interaction de qualité au travers d'échanges vocaux accordés. (+)

- [ ] Les échanges vocaux sont souvent adaptés, permettant une interaction et un échange vocal accordés, parfois artificiels ou exagérés. L'un des deux partenaires peut être plus actif dans la mise en place et dans l'expression des échanges vocaux. (+/-)

- [ ] Les échanges vocaux permettent parfois une interaction de qualité et sont majoritairement inadaptés : ils sont ambivalents, pas spontanés, sur un versant négatif (colère, enfant grincheux, etc.), brefs, mécaniques, inconsistants et viennent uniquement en réponse à une stimulation ou une sollicitation. La qualité des échanges vocaux est pauvre. (-/+)

- [ ] Aucun échange vocal durant l'interaction : absence d'expression vocale sur la majorité ou sur la totalité de l'interaction. (-)

3. Position et contacts corporels

☐ La position des corps permet une interaction réciproque et recherchée par les deux partenaires. La capacité d'ajustement posturale est toujours réalisée dans le but de maintenir l'interaction. Les contacts corporels sont adaptés, affectueux, chaleureux, tendres et signes de plaisir manifestes dans l'interaction. Bon contact et bonne qualité. (+)

☐ Le positionnement des corps est souvent à bonne distance et adapté, les contacts corporels peuvent être chaleureux. La position des corps peut être adaptée pour seulement l'un des deux partenaires (par exemple, lors d'un jeu, le bébé peut être confortablement installé alors que le parent ne va pas l'être). Le réajustement peut se faire et souligner la recherche active d'une interaction. (+/-)

☐ La position des corps permet l'interaction à minima avec parfois un ajustement corporel de l'un ou de l'autre des partenaires. Le contact corporel peut se faire sur un versant distant ou proche (collage). La proximité n'est pas chaleureuse. Le contact corporel est majoritairement inadapté, difficile, avec peu de plaisir. Contact présent mais pas de bonne qualité. (-/+)

☐ La position des corps ne permet pas l'interaction. Les visages sont difficilement accessibles. Le contact corporel est fui par les deux partenaires. Les deux partenaires marquent une distance inappropriée. Il n'y a pas de contact. (-)

4. Expression des affects

☐ Les affects sont adaptés, modulés, positifs et spontanés. La richesse des affects exprimés est en adéquation entre les deux partenaires. Les affects physiques, visuels et vocaux sont chaleureux (caresses, joie, murmures, sourires et tendresse). (+)

☐ La gamme d'expression des affects est plus variée et peut être positive, mais pas toujours en adéquation entre les deux partenaires. L'un des deux peut avoir une expression d'affect plus gaie, modulée, spontanée et variée, par rapport à l'autre. L'accordage dans l'expression des affects des deux partenaires peut être fluctuant (expression d'affects positifs, mais brusquement interrompue, retardée, en décalage avec le comportement de l'autre). Le visage est parfois en adéquation avec la situation, mobile et expressif. (+/-)

☐ Les affects exprimés sont pauvres, généralement sur un versant négatif (colère, frustration continue…) et en inadéquation entre les deux partenaires. L'expression des affects est majoritairement inadaptée et désaccordée. Une absence de plaisir ou d'expression affective chaleureuse est à noter. Pas de mobilité faciale spontanée ou de changements fréquents d'émotions (sourires figés et inadaptés). (-/+)

☐ Aucune expression perceptible d'affect et de plaisir dans l'interaction : visage figé, impassible, inexpressif, triste. Indifférence des deux partenaires. Aucune chaleur lors de l'interaction. La gamme d'affects exprimés est nulle et non variable. (-)

5. Engagement et réciprocité des échanges

- ▢ L'échange est toujours adapté, avec une alternance réciproque. L'attention conjointe et les mouvements d'allers et retours entre les deux partenaires sont fluides et au cœur de l'interaction. (+)

- ▢ Les deux partenaires s'engagent souvent dans des échanges de qualité, avec parfois présence de silences inadaptés. L'attention conjointe est quelquefois présente et accordée, avec des mouvements d'allers et retours entre les deux partenaires. (+/-)

- ▢ Le parent ne réagit que peu aux initiatives d'échange de son enfant et/ou l'enfant réagit peu aux initiatives du parent. Peu d'allers et retours entre les partenaires, difficulté dans l'initiation ou le maintien du tour de rôle (longues pauses vides). L'attention conjointe est rarement possible. (-/+)

- ▢ Aucune réciprocité des échanges possible. L'échange ou le dialogue n'est initié par aucun des partenaires. L'échange ou le dialogue n'est pas engagé par l'un ou par l'autre des partenaires. Les partenaires ne s'impliquent pas dans l'échange. (-)

6. Présence parentale aidante - Sensibilité maternelle/parentale

- ▢ Le parent observe les signaux* de son enfant dans diverses circonstances et y est sensible. Il peut interpréter justement les signaux de son enfant, peut faire la distinction entre ses propres besoins et ceux de son enfant. La réponse du parent aux besoins de son enfant est constante, il peut faire le lien entre ses comportements et les réactions de son enfant. L'aide est adaptée aux sollicitations et difficultés constatées de l'enfant, le parent peut adapter sa distance dans l'aide fournie et mesurer le degré d'intervention nécessaire. (+)

- ▢ Le parent détecte souvent les signaux* de son enfant mais en ignore d'autres. Il interprète de manière adaptée la plupart des signaux de son bébé, mais parfois en fonction de ses propres besoins et/ou en lien avec son état émotionnel. L'aide apportée à l'enfant est parfois en décalage avec ce que l'enfant émet comme signaux. Le parent peut être présent, aidant mais parfois maladroit. (+/-)

  * Signaux : regards, pleurs, cris, enfouissement, babillage, pointage, évitement…

- ▢ Le parent détecte certains signaux* mais les interprète majoritairement mal ou en lui attribuant de mauvaises intentions. Il peut réagir aux signaux de son enfant de manière excessive et chaotique. Il offre des soins généralement mécaniques et sans interaction avec son bébé (nourrit son enfant même si celui-ci montre qu'il n'a plus faim…). Le parent fournit une aide insuffisante au vu des sollicitations de son bébé. (-/+)

- ▢ Le parent ne détecte pas les signaux* de son bébé, n'arrive pas à les interpréter et n'a aucune réponse à apporter aux signaux de son enfant, aucune aide fournie. (-)

7. Intrusivité du parent

- [ ] Parent qui ne présente pas d'intrusivité, ni d'action contrôlante. (+)

- [ ] Parent parfois intrusif dans l'interaction. Le contrôle qu'il exerce peut être fluctuant et parfois accordé aux besoins de son enfant. (+/-)

- [ ] Parent majoritairement intrusif dans l'interaction. Le contrôle exercé par le parent ne se fait pas en accordage avec l'enfant. (-/+)

- [ ] Parent intrusif sur la totalité de l'interaction. Le parent fait brusquement irruption dans l'espace de l'enfant, peut le manipuler contre sa volonté ou saisir brusquement ses jouets lorsqu'il les utilise (ou lui en imposer des nouveaux). Le parent exerce un contrôle omnipotent malgré les signes de désapprobation de l'enfant. (-)

8. État subjectif induit par l'observation de l'enfant dans l'interaction

- [ ] J'ai une sensation agréable, je me sens détendu(e), la relation est plaisante à observer, j'éprouve de l'intérêt à observer l'enfant en interaction avec son parent. (+)

- [ ] J'ai un sentiment neutre en observant l'enfant, ni plaisir ni indifférence, j'ai un intérêt pour l'enfant en interaction avec son parent mais avec moins de plaisir que décrit dans l'item ci-dessus. (+/-)

- [ ] Je ressens un léger sentiment de malaise progressif en observant l'enfant dans l'interaction. (-/+)

- [ ] Je me sens confus(e), l'observation de l'enfant dans l'interaction est éprouvante, sentiment de malaise d'emblée perceptible. (-)

9. État subjectif induit par l'observation du parent dans l'interaction

- [ ] J'ai une sensation agréable, je me sens détendu(e), la relation est plaisante à observer, j'éprouve de l'intérêt à observer le parent en interaction avec son enfant. (+)

- [ ] J'ai un sentiment neutre en observant le parent, ni plaisir ni indifférence, j'ai un intérêt pour le parent en interaction avec son enfant mais avec moins de plaisir que décrit dans l'item ci-dessus. (+/-)

- [ ] Je ressens un léger sentiment de malaise progressif en observant le parent dans l'interaction. (-/+)

- [ ] Je me sens confus(e), l'observation du parent dans l'interaction est éprouvante, sentiment de malaise d'emblée perceptible. (-)

Observations

Calculez le nombre de points en fonction des signes associés à la réponse que vous avez choisie.

(+) = 3

(+/-) = 2

(-/+) = 1

(-) = 0

Note globale :

Score	Qualité d'interaction	Recommandations
</= 15	Mauvaise	Intervention et orientation recommandée
16 - 20	Moyenne	Surveillance recommandée
>20	Bonne qualité	

Si vous utilisez l'échelle, merci de référencer la revue *PLOS ONE journal.* Vous pouvez nous faire des retours dans un but d'amélioration via : [secretariat.pedopsychiatrie-brabois@cpn-laxou.com].

When using this scale, please reference the PLOS ONE journal. Feedback for improvement purposes can be sent to: [secretariat.pedopsychiatrie-brabois@cpn-laxou.com].

Échelle créée par Dr. Sophie BUCHHEIT et Pr. Fabienne LIGIER

Scale developed by Dr. Sophie BUCHHEIT and Professor Fabienne LIGIER
