# Supplementary material for: Development and validation of the MIPPE: A novel dyadic assessment tool for early parent-child interactions in clinical practice
Source: PLoS One. 2026 Apr 24;21(4):e0347521. doi: 10.1371/journal.pone.0347521 (PMC13108784; doi:10.1371/journal.pone.0347521)
Supplement: S2 File — English translation of form for collecting consent to the use of the participant’s image and that of their child in the context of the PERL project. (PDF) [file pone.0347521.s002.pdf]

# To evaluate the effects of home-based preventive support on the development of young children
# PERL: Early Childhood, Research-Action in Lorraine

Nancy Psychotherapy Center
1, rue du Docteur Archambault
BP11010
54521 LAXOU Cedex
Phone : 03.83.92.50.50

Private label Lunéville
Service de PMI
28, rue de la République
54300 LUNEVILLE
Phone : 03.83.74.44.24

Project coordinator: Sophie BUCHHEIT
Courriel : sophie.buchheit@cpn-laxou.com

## Form for collecting the person's consent to the use of his or her image and that of his or her child

### Identification of the child's legal representative (filmed)

**Name** : **Maiden Name** :
**First name** :
**Address** :
**Date and place of birth** : **the** **at**

Taking into account the written and/or oral information relating to the project described on page 2, I declare that I consent to the use of my image under the restrictive conditions detailed below and that I have received a copy of this document.
**Possible remarks** :

**To the** **Signature**

### Child identification

**Name** :
**First name** :
**Date and place of birth** : **the** **at**

Taking into account the written and/or oral information relating to the project described on page 2, I declare that I confirm the consent of my child named above to the use of his or her image under the limiting conditions detailed below and that I have received a copy of this document.
**Possible remarks** :

**Has** **the** **Signature**

**<u>Project Information</u>**

The video is part of the PERL research project on baby development. It will be used during home visits to complete observations on the baby. The family will be able to receive a copy of the videos upon request.

Permission to use my image and that of my child in the context of this project is given to: **Mrs. Sophie BUCHHEIT, clinical psychologist** and her team under the following restrictive conditions and for the following uses:

- **Type of Delivery** :
    - □ Television Broadcasting
    - □ Multimédia (internet, intranet,…).
    - □ Video
    - □ Other
        - ■ Possible clarifications: no distribution

- **Use** :

Commercial □ (if commercial use, financial implications to be specified for the sponsor and patients).

■ Non-commercial

- **Scope of use** :
    - ■ Use in an educational setting
    - ■ Use in seminars or conferences
    - □ Use in video production competitions
    - ■ Other

        Possible details: use in the context of research evaluation

- **Places where any copies of the image medium are kept** : under lock and key, at the CMP for children and adolescents in Vandoeuvre les Nancy.

This authorization will take effect from **the date of signature of this form and will remain valid for an unlimited period of time unless you object. Participants in the PERL project - give their consent to the use of their image under the conditions defined above - may withdraw this consent at any time from Mrs. Sophie BUCHHEIT, clinical psychologist, coordinator of the PERL project.**

Comments and images must not infringe on the reputation or privacy of the participants.

**Name, date and signature of the custodian of consent:**
