# Supplementary material for: Development and validation of the MIPPE: A novel dyadic assessment tool for early parent-child interactions in clinical practice
Source: PLoS One. 2026 Apr 24;21(4):e0347521. doi: 10.1371/journal.pone.0347521 (PMC13108784; doi:10.1371/journal.pone.0347521)
Supplement: S3 File — (PDF) [file pone.0347521.s003.pdf]

Evaluer les effets d'un accompagnement préventif à domicile sur le développement du jeune enfant
PERL : Petite Enfance, Recherche-action en Lorraine

Centre Psychothérapique de Nancy
1, rue du Docteur Archambault
BP11010
54521 LAXOU Cedex
Téléphone : 03.83.92.50.50

MDD Lunéville
Service de PMI
28, rue de la République
54300 LUNEVILLE
Téléphone : 03.83.74.44.24

Coordinatrice du projet : Sophie BUCHHEIT
Courriel : sophie.buchheit@cpn-laxou.com

Formulaire de recueil du consentement de la personne à l'utilisation de son image et celle de son enfant

Identification du représentant légal de l'enfant (filmé)

Nom : **Nom de jeune fille** :
Prénom :
Adresse :
Date et lieu de Naissance : **le** **à**

Compte-tenu des informations écrites et/ou orales relatives au projet décrit page 2, je déclare consentir à l'utilisation de mon image aux conditions limitatives détaillées ci-après et avoir reçu un exemplaire du présent document.
Remarques éventuelles :

A **le** **Signature**

Identification de l'enfant

Nom :
Prénom :
Date et lieu de Naissance : **le** **à**

Compte tenu des informations écrites et/ou orales relatives au projet décrit page 2, je déclare confirmer le consentement de mon enfant désigné ci-dessus à l'utilisation de son image aux conditions limitatives détaillées ci-après et avoir reçu un exemplaire du présent document.
Remarques éventuelles :

A **le** **Signature**

Information relative au projet

La vidéo s'inscrit dans le cadre du projet de recherche PERL sur le développement du bébé. Elle sera utilisée lors de visites à domicile pour compléter les observations sur le bébé. La famille pourra recevoir une copie des vidéos si elle en fait la demande.

L'autorisation d'utiliser mon image et celle de mon enfant dans le cadre du présent projet est donnée à [a] : **Madame Sophie BUCHHEIT, psychologue clinicienne** et son équipe aux conditions limitatives et pour les usages ci-dessous :

- **Type de diffusion** :

  □ Télédiffusion
  □ Multimédia (internet, intranet,…).
  □ Vidéo
  □ Autre
    ■ Précisions éventuelles : pas de diffusion

- **Utilisation** :

  □ Commerciale (si utilisation commerciale, implications financières éventuelles à préciser pour le promoteur et les patients).
  ■ Non commerciale

- **Cadre de l'utilisation** :

  ■ Utilisation dans un cadre pédagogique
  ■ Utilisation en séminaires ou conférences
  □ Utilisation dans le cadre de concours de productions vidéo
  ■ Autre
    Précisions éventuelles : utilisation dans le cadre de l'évaluation de la recherche

- **Lieux de conservation d'éventuels exemplaires du support de l'image** : sous clé, au CMP enfants et adolescents de Vandoeuvre les Nancy.

La présente autorisation prendra effet à compter de **la date de signature du présent formulaire et restera valable sans limitation de durée sauf opposition de votre part. Les participants au projet PERL - donnent leur consentement à l'utilisation de leur image dans les conditions définies plus haut – peuvent à tout moment retirer ce consentement auprès de Mme Sophie BUCHHEIT, psychologue clinicienne, coordinatrice du projet PERL.**

Les commentaires et images ne devront pas porter atteinte à la réputation ou à la vie privée des participants.

Nom, date et signature du dépositaire du consentement :

[a] Nom et fonction du professionnel chargé du projet
