# Supplementary material for: Development and validation of the MIPPE: A novel dyadic assessment tool for early parent-child interactions in clinical practice
Source: PLoS One. 2026 Apr 24;21(4):e0347521. doi: 10.1371/journal.pone.0347521 (PMC13108784; doi:10.1371/journal.pone.0347521)
Supplement: S4 File — English translation of informed consent form for participant inclusion in the PERL study. (PDF) [file pone.0347521.s004.pdf]

To evaluate the effects of home-based preventive support on the development of young children
PERL: Early Childhood, Action Research in Lorraine

CONSENT FORM

Mrs. Sophie BUCHHEIT, coordinator of the project, offered me to participate in the research entitled "**PERL: Early Childhood: Research-action in Lorraine**", organized by the Protection Maternelle Infantile du Lunévillois and the Psychotherapy Center of Nancy and of which **Professor KABUTH** is medical director.

I have noted the contact details of the professional in charge of the study whom I can contact **:**
Sophie BUCHHEIT, coordinating psychologist 06.48.60.31.90

Research sponsor: **Nancy Psychotherapy Center**
Address: **1 rue du Docteur Archambault- BP 11010- 54521 LAXOU Cedex**

I, the undersigned,
.... *(first and last name)* as mother
....*(surname and first name)* as father check here if there is only one person with parental authority ☐
declares that he has understood the purpose and modalities, the course and the duration of this research, which were fully explained to me by Sophie BUCHHEIT, coordinator of the project.
I accept that my child .... *(first and last name)* participates in this research
under the conditions specified in the attached backgrounder.

I have been informed of the purpose of this research, how it will be carried out and what my participation will mean for me.

I have read the information document: version N°5 of 23/06/2023. I understood this information and got the answers to the questions I asked. I had time to reflect on my participation in this action research.

I have been told that I am free to accept or refuse, and that my participation in this action research is voluntary.

I have been informed that in accordance with the Clinical Research Regulations:
- The Committee for the Protection of Persons North West IV gave a favourable opinion for the realization of this research on 14/11/2017.
- The French National Agency for the Safety of Medicines and Health Products has been informed of the implementation of this study
- the Commission Nationale de l'Informatique et des Libertés gave its authorization to carry out this research on 30/01/2018.

If I wish, I will be informed of the overall results of the study and whether they have been published under the conditions set out in the information document. If the results are published in a medical or scientific journal, my identity will not be revealed, nor that of my child.

I am fully aware that I can withdraw my consent to my participation in this research at any time without having to justify myself, regardless of my reasons and without incurring any responsibility or prejudice to the professionals involved in the research. In this case, the data previously collected will be kept unless I refuse (in this case, it will be deleted only if this does not compromise the results of the search).

My consent does not relieve the investigator and the sponsor of all their responsibilities and I retain all my rights guaranteed by law.

I have been informed that as part of the Research in which I am participating, my personal data will be processed to enable the results of the research to be analysed in relation to its objective, under conditions guaranteeing their confidentiality.

I accept that my data, recorded during this research, may be subject to automated processing by the promoter on its behalf. I have noted that I may at any time exercise my right of access, rectification, opposition, limitation, deletion and data portability provided for by the European Data Protection Regulation with the Data Protection Officer of the Nancy Psychotherapy Center, whose contact details are specified in the information note I received.

In the event of a disagreement, I may also file a complaint with the CNIL.

À compléter par le ou les parents (ayant l'autorité parentale):

**Mère**	**et/ou**	**Père**
**Nom :**		**Nom :**
**Prénom :**		**Prénom :**

J'accepte **librement et volontairement** de participer à cette Recherche-action. Je conserverai un exemplaire de la lettre d'information et du formulaire de consentement dûment complétés et signés.

**Date et signature**	**Date et signature**

Sophie BUCHHEIT
Research Coordinating Psychologist

Date and signature

On 04/09/2023

Sophie Buchheit
