# Supplementary material for: Development and validation of the MIPPE: A novel dyadic assessment tool for early parent-child interactions in clinical practice
Source: PLoS One. 2026 Apr 24;21(4):e0347521. doi: 10.1371/journal.pone.0347521 (PMC13108784; doi:10.1371/journal.pone.0347521)
Supplement: S5 File — (PDF) [file pone.0347521.s005.pdf]

Evaluer les effets d'un accompagnement préventif à domicile sur le développement du jeune enfant
PERL : Petite Enfance, Recherche-action en Lorraine

FORMULAIRE DE CONSENTEMENT

Madame Sophie BUCHHEIT, coordinatrice du projet, m'a proposé de participer à la Recherche intitulée «**PERL : Petite Enfance : Recherche-action en Lorraine**», organisée par la Protection Maternelle Infantile du Lunévillois et le Centre Psychothérapique de Nancy et dont le **Professeur KABUTH** est responsable médical.

J'ai bien noté les coordonnées du professionnel en charge de l'étude que je pourrai contacter **:**
Sophie BUCHHEIT, psychologue coordinatrice 06.48.60.31.90

Promoteur de la recherche : **Centre Psychothérapique de Nancy**
Adresse : **1 rue du Docteur Archambault- BP 11010- 54521 LAXOU Cedex**

Je soussigné(e),
…... *(nom et prénom)* en qualité de mère
………………………………………………….*(nom et prénom)* en qualité de père
cocher ici s'il n'existe qu'un seul titulaire de l'autorité parentale ☐
déclare avoir compris le but et les modalités, le déroulement et la durée de cette recherche, qui m'ont été pleinement expliqués par Sophie BUCHHEIT, coordinatrice du projet.
J'accepte que mon enfant …... *(nom et prénom)* participe à cette recherche dans les conditions précisées dans le document d'information ci-joint.

J'ai été informé(e) de l'objectif de cette recherche, de la façon dont elle va être réalisée et de ce que ma participation va impliquer pour moi.

J'ai bien lu le document d'information: version N°5 du 23/06/2023. J'ai compris ces informations et j'ai obtenu les réponses aux questions que j'ai posées. J'ai eu le temps de réfléchir à ma participation à cette recherche-action.

Il m'a été précisé que je suis libre d'accepter ou de refuser, et que ma participation à cette recherche-action est volontaire.

J'ai été informé(e) que conformément à la réglementation sur les recherches cliniques :
- le Comité de Protection des Personnes Nord Ouest IV a rendu un avis favorable pour la réalisation de cette recherche en date du 14/11/2017.
- l'Agence Nationale de Sécurité du Médicament et des produits de santé a été informée de la mise en œuvre de cette étude
- la Commission Nationale de l'Informatique et des Libertés a donné son autorisation pour la réalisation de cette recherche en date du 30/01/2018.

Si je le souhaite, je serai informé(e) des résultats globaux de l'étude et s'ils ont fait l'objet d'une publication dans les conditions exposées dans le document d'information. En cas de publication des résultats dans une revue médicale ou scientifique, mon identité ne sera pas révélée, ni celle de mon enfant.

Je suis parfaitement conscient(e) que je peux à tout moment retirer mon consentement à ma participation à cette recherche sans avoir à me justifier et cela quelles que soient mes raisons et sans encourir aucune responsabilité ni aucun préjudice avec les professionnels impliqués dans la recherche. Dans ce cas, les données préalablement recueillies seront conservées sauf refus de ma part (dans ce cas, elles seront supprimées uniquement si cela ne compromet pas les résultats de la recherche).

Mon consentement ne décharge en rien l'investigateur et le promoteur de l'ensemble de leurs responsabilités et je conserve tous mes droits garantis par la loi.

J'ai été informé(e) que dans le cadre de la Recherche à laquelle je participe, un traitement de mes données personnelles va être mis en œuvre pour permettre d'analyser les résultats de la recherche au regard de son objectif, dans les conditions garantissant leur confidentialité.

J'accepte que mes données, enregistrées à l'occasion de cette recherche, puissent faire l'objet d'un traitement automatisé par le promoteur pour son compte. J'ai bien noté que je pourrai à tout moment faire valoir mon droit d'accès, de rectification, d'opposition, de limitation, d'effacement et de portabilité des données prévu par le Règlement Européen sur la Protection des Données auprès du Délégué à la Protection des Données du Centre Psychothérapique de Nancy, dont les coordonnées sont précisées dans la note d'information que j'ai reçue.

En cas de désaccord, je pourrai également effectuer une réclamation auprès de la CNIL.

À compléter par le ou les parents (ayant l'autorité parentale):

**<u>Mère</u>**	**et/ou**	**<u>Père</u>**
**Nom :**		**Nom :**
**Prénom :**		**Prénom :**

J'accepte **librement et volontairement** de participer à cette Recherche-action. Je conserverai un exemplaire de la lettre d'information et du formulaire de consentement dûment complétés et signés.

Date et signature **Date et signature**

Sophie BUCHHEIT
Psychologue coordinatrice de la recherche

Date et signature

Le 04/09/2023

Sophie Buchheit
