# Supplementary material for: Development and validation of the MIPPE: A novel dyadic assessment tool for early parent-child interactions in clinical practice
Source: PLoS One. 2026 Apr 24;21(4):e0347521. doi: 10.1371/journal.pone.0347521 (PMC13108784; doi:10.1371/journal.pone.0347521)
Supplement: S6 File — English translation of complete research protocol of the PERL program (Petite Enfance Recherche-action en Lorraine), including all amendments. (PDF) [file pone.0347521.s006.pdf]

To evaluate the effects of home-based preventive support on the development of young children
PERL: Early Childhood, Research-Action in Lorraine

Nancy Psychotherapy Center
1, rue du Docteur Archambault
BP11010
54521 LAXOU Cedex
Phone : 03.83.92.50.50

Private label Lunéville
Service de PMI
28, rue de la République
54300 LUNEVILLE
Phone : 03.83.74.44.24

Project coordinator: Sophie BUCHHEIT
Email: sophie.buchheit@cpn-laxou.com

PERL RESEARCH PROTOCOL
including substantial amendment N°7

Title
To evaluate the effects of home-based preventive support on the development of young children
PERL : Early Childhood : Action Research in Lorraine

Promoter
Centre Psychothérapique de Nancy, 1 rue du Docteur Archambault, BP11010 – 54 521 Laxou Cedex

Coordinating Investigator
Sophie BUCHHEIT

Protocol version
Version 9 of 23/06/2023 including substantial amendment N°7, having received a favourable opinion from the CPP Nord Ouest IV on 24/08/2023.

Initial favourable opinion of the CPP Nord Ouest IV on 14/11/2017
Favourable opinion of the CPP Nord Ouest IV for the substantial modification N°1 on 25/01/2018 Favourable opinion of the CPP Nord Ouest IV for the substantial modification N°2 on 20/12/2018 Favourable opinion of the CPP Nord Ouest IV for the substantial modification N°3 on 23/04/2019 Favourable opinion of the CPP Nord Ouest IV for the substantial modification N°4 on 26/09/2019 Favourable opinion of the CPP Nord Ouest IV for the substantial modification N°5 on 27/05/2021 Favourable opinion of the CPP Nord Ouest IV for the substantial modification N°6 on 18/08/2022

Study Type
Interventional research with minimal risks and constraints, mentioned in 2° of Article L.1121-1 of the Public Health Code.

Number ID RCB
2017-A00896-47

1. INTRODUCTION

1.1 Rationale/Background

To fight against social inequalities in health (which is one of the priorities of health policy in France), early childhood is a key period. In this context, parenting support is recognized as a potentially effective lever (in *World Health Organization. Closing the gap in a generation: health equity through action on the social determinants of health. Final Report [Internet]. Geneva: Commission on Social Determinants of Health; 2008, p. 256. ; Guyer B, Ma S, Grason H, Frick KD, Perry DF, Sharkey A, et al. Early Childhood Health Promotion and Its Life Course Health Consequences. Acad Pediatr. 2009 May; 9(3):142–149.e71. ; Barlow J, Smailagic N, Huband N, Roloff V, Bennett C. Group- based parent training programmes for improving parental psychosocial health. In: Cochrane Database of Systematic Reviews [Internet]. John Wiley & Sons, Ltd; 2014 [cited 2016 Jan 8]. ; Barlow J, Smailagic N, Bennett C, Huband N, Jones H, Coren E. Individual and group based parenting programmes for improving psychosocial outcomes for teenage parents and their children. In: Cochrane Database of Systematic Reviews [Internet]. John Wiley & Sons, Ltd; 2011 [cited 2016 Feb 24]. ; Shonkoff JP, Boyce WT, McEwen BS. Neuroscience, Molecular Biology, and the Childhood Roots of Health Disparities: Building a New Framework for Health Promotion and Disease Prevention. JAMA. 2009 Jun 3; 301(21):2252. ; Postnatal parental education for optimizing infant general health and parent-infant relationships. Cochrane Database Syst Rev. 2013 Nov*) et les actions d'accompagnement à la parentalité sont promues.

More specifically in the Lunéville region, a significant frequency of language delays is observed each year in 4-year-old children on the ERTL4 test, systematically carried out by the PMI in kindergarten. These figures reveal a context of fragility and precariousness of families and encourage us to offer support as early as possible.

An action research project (Interreg project) offered to children born in 2010 and their parents in the areas of Blâmont, Cirey-Sur-Vezouze and Badonviller showed that home visits carried out by a psychologist (in *Taking care of the baby and his family: an action-research experience in perinatality / Fidry Elise ; Claudon Philippe; Saad Saint-gilles Stéphanie ; Sibertin Blanc Daniel. Neuropsychiatry of Childhood and Adolescence, 2014, No. 3; From first glances to first words: a Lorraine experience of perinatal prevention / Fidry Elise ; Sibertin Blanc Daniel ; Claudon Philippe. -Paris: L'Harmattan, 2014. - 65-83*) significantly **reduced developmental and language delays**, with:

1) 39% delays in the developmental test at 24 months in the intervention cohort compared to 74% delays in the control cohort;
2) 21% delays in the language test at 4 years in the intervention cohort compared to 31% in the control cohort;
3) Need for care (speech therapy or psychological) for 23% of children in the intervention cohort compared to 49% of control children.

The objective of the PERL project is to use this model of home support and to adapt it to offer it to a wider sector, while integrating it into existing practices, within the framework of a partnership between Maternal and Child Protection and child psychiatry. An innovative prevention system to support interactions and

will be offered to about sixty families, randomly recruited from the general population. If the family agrees to participate, they will then benefit from regular home visits by a childcare worker accompanied by a psychologist for a period of 4 years from the birth of the child. This home support, focused on listening and observation, is based on three axes: the baby's development, parenthood, parent-child interaction. In conjunction with the practical implementation of this system, we propose to evaluate it according to a pre-established methodological plan.

This research-action is part of a strong partnership dynamic and is supported by several institutions: Departmental Council of Meurthe et Moselle, Nancy Psychotherapy Centre, Grand-Est Regional Health Agency, Meurthe et Moselle Family Allowances Fund.

1.2 Main Objective of PERL Research

To assess the effects of preventive support at home by an early childhood professional on the prevalence of language and developmental delays in children compared to usual care.

1.3 Secondary Objectives of PERL Research

1- To evaluate longitudinally: the development of the child up to the age of 4, the quality of parent-child interactions and the experience of parenthood.
2- To evaluate longitudinally the experience of home support.

2. METHODOLOGICAL ASPECTS

The cohorts will be subjected to a longitudinal and comparative intergroup evaluation.

2.1 Population

It is planned to include:
- 64 families for the "intervention" group;
- 64 families for the "control" group.

A power calculation was performed: If we start with one case for a control, with a power of 80%, an alpha risk of 5%, a prevalence of developmental delay of 50% in controls and 25% in patients benefiting from the device, we obtain a result of: 58 people in the intervention group and 58 people in the control group. Taking into account a 10% loss of subjects during the research, we obtain 64 subjects to be recruited for the intervention group and 64 subjects for this action research.
In addition, the figure of 64 has also been estimated to meet the constraints of the practical context of childcare workers, which conditions the limit of subjects that can be treated. The childcare workers continue their usual activity in PMI, to which are added VADs (home visits) for PERL research. It therefore seemed relevant to us, in view of the continuation of their professional activity, and after discussion with the Departmental Council team (PMI service), to allocate 8 families per childcare worker. Initially, we had agreed to follow up with 8 families per childcare worker, after discussion with the territorial doctor (PMI). Following the implementation of the first VAD carried out by the childcare workers, they have

informed that the maximum number of families with whom they could set up a follow-up for 4 years was 4 per childcare worker. In order to get our 64 families for the "intervention group", we have expanded the recruitment area of the study (from the Pays Lunévillois to the southern Meurthe et Moselle) in order to be able to involve more childcare workers (6 additional childcare workers) and disperse the workload.

This research will be offered to parents living in the Meurthe et Moselle Sud sector who have given birth to a child between 2018 and 2020. There are therefore two criteria for inclusion:

- birth of the child in the years 2018 to 2020;
- place of residence (Meurthe and Moselle Sud).

There are two criteria for non-inclusion:

- The families do not speak French, the language barrier does not allow the clear understanding of the items of some tests and makes it difficult to communicate with the childcare worker.
- Travellers who are not sedentary, who will not be able to benefit from VAD from birth to 4 years or VAD every 4 and 24 months

For this research, we have chosen to work with the general population and not to recruit families according to vulnerability criteria. This will allow us to avoid stigmatizing families and focus on their abilities.

In addition, the initial recruitment for the PERL study was limited to the Lunéville sector. This sector was chosen in order to be as close as possible to the geographical area of the first INTERREG research. The initial request to set up the PERL research was, in the first place, a professional approach by the territorial doctor of the Pays Lunévillois (PMI). We have chosen to extend this perimeter of recruitment and follow-up of families to the Meurthe and South Moselle sector because the lack of human resources (PMI professionals) in the Lunévillois territory leads to an excessive workload for the childcare workers who follow the families included in the "intervention group". In order for the inclusion period to remain within a reasonable timeframe and consistent with our scientific objectives, we felt it necessary to extend the scope of recruitment so that childcare workers from other sectors could intervene in the PERL study (substantial modification N°4 to the protocol). The extension to the Meurthe et Moselle Sud makes it possible to maintain an equivalent territory in terms of population characteristics (results of the language development test and population profile) and not to create bias in the subsequent analysis of the data collected.

2.2 Procedures for recruiting families

2.2.1 Systematic mail

Families who have declared their pregnancy and whose due date is expected between September 2018 and December 2020 will all receive a letter informing them that the PERL research began in 2018 and that they are likely to be contacted to offer them to participate.

Following this letter, families can contact us to oppose this approach by simple phone call, email or post. If they do not issue any refusals, they will be included in the list with which we will randomly recruit to determine which families are part of the "control" cohort and which are part of the cohort

"intervention". Once the families have been contacted, all the data will be anonymised in a systematic way.

The "control" cohort will therefore be selected in the same territory and according to a random recruitment identical to the "intervention" cohort.

2.2.2 Random recruitment

All families participating in the study will be recruited on a voluntary basis. They will be selected by random recruitment (Zelen randomization) carried out every month in order to reach the expected number of inclusions (64 interventions and 64 controls). It was agreed, in agreement with the scientific research committee, to select the first eight families drawn at random and agreeing to participate in the research each month until reaching 8 inclusions among the people benefiting from the intervention (one family per month per childcare worker) and 8 inclusions among the controls per month.

2.2.3 First information by mail

Randomly recruited families will be informed that they are concerned by the PERL action research around the 8th month of pregnancy so as not to include preterm births (which would constitute a bias in the interpretation of quantitative data collected during certain developmental tests). A letter will be sent to their homes, accompanied by a brochure presenting the action research. An appointment with the coordinating psychologist will be offered to them at their home or in an early childhood structure, at their convenience. This meeting will make it possible to transmit to parents information concerning the PERL action research without this committing them to participate.

2.2.4 Information given at the first meeting

If this first meeting is accepted by the parents, it will take place during the eighth month of pregnancy, in order to present this research to them. Detailed information will be provided by the coordinator concerning the practical arrangements (number and frequency of home visits, content of exchanges, persons present, duration,...), their rights (to stop the search, to retrieve data concerning them,...), and data processing (anonymity, global and not individual conclusions). This exchange will be supported by an information note that will then be given to them, accompanied by a consent form. They will then have a period of reflection to decide whether they wish to be part of the PERL action research.
If they agree, the consent form and briefing note will be signed, and they will be considered included in the research.

2.3 Preventive support model

The paediatric nurses and the coordinating psychologist benefited from a 5-day training period (35 hours) before starting the project around home observation for preventive purposes. This training combined practice and theory to prepare for action in the field. It was delivered by Elise Fidry, the psychologist in charge of the previous research and by Dr. Stéphanie Saad Saint-Gilles. This training was completed one year before the start of the

place of action, and being difficult to reproduce to future childcare workers who would join the system, we have agreed to reorganize the training methods. From now on, the childcare workers participating in the PERL system will benefit every semester (to be reassessed according to the needs of the childcare workers according to their request) from working days and exchanges and continuous training at the rate of one hour per month, after supervision (see below). The future childcare workers will have an interview with the psychologist coordinating the project in order to explain the modalities of the study and to present the model and protocol concerning Home Visits.

The preventive support model provides that home visits are structured around three stages: joint observation of the baby with his parents, interview with the parents and interaction, in particular through play.

The support is based on three main axes:
- Support for parenthood: the active and attentive listening of the professional aims to promote the emergence of imaginary, fantastical and narcissistic parental representations of the baby. Parenthood is to be considered as a maturative process whose temporality is singular for each individual. Access to parenthood implies a set of identity and relational reshuffles that can be supported in the context of the meetings.
- Observation of the baby with his parents: the time spent observing the baby and his progress allows the parent to identify with this "observing function" and thus promote the development of their toddler.
- Promote parent-baby interaction: as attachment theory has demonstrated, at birth, the baby is a social being for whom interactive exchange with a reassuring figure represents a primary need. In the accompaniment, it seems important to take an interest in the progressive construction of attachment bonds and to invite the parents to enter into a relationship with the baby in care or through play. The professional can also strengthen the security of the bond by offering, thanks to the framework of action research, regularity and a secure attachment to the parents.

The accompaniment is inspired by Bick's (1963) model of home observation, which insists on the essential aspects of the observer's position: "listening", "receptivity", "conscious and unconscious attention". It also draws on the contributions of Fraiberg's (1989) child mental health program, which values careful observation, empathetic listening, a nurturing care relationship with parents, and interest in past experiences. The professional is present to the baby and his parents in continuity and ensures a countenance, as described by Bion (1962). He is attentive to the baby's development, early interactions and the experience of parenthood.

The team of paediatric nurses will benefit from two hours of monthly supervision carried out by Sophie BUCHHEIT, psychologist coordinating the research. The purpose of these times will be to analyse and elaborate observations made at home. Supervision times will be flexible according to the working conditions of the childcare workers, the evolution of the health conditions that we have to face and adapt. In the context of research, the essential thing is to be able to adapt to the field, to the professionals in the field, while maintaining the framework of supervision as mandatory within the framework of the system. Childcare workers can also have access to individual supervision times once a week, provided by Sophie BUCHHEIT. It is also specified to the childcare workers that they can reach the coordinating psychologist on her personal phone at any time in order to discuss the follow-up of the families met. Those

two hours of supervision will be followed by an exchange focused on the processes at work during the meetings and the theoretical-clinical contributions that S. BUCHHEIT (who is writing a doctoral thesis in psychology on this research) can bring. In this way, the training of paediatric nurses will continue in parallel with the VADs, and the themes addressed can be enhanced with concrete clinical cases concerning the follow-up of PERL families.

2.4 Rhythm of home visits

As part of this research, families will benefit from:

➢ *For "intervention" families*

- Home visits by a childcare worker which will focus on three times: a time to observe the baby's development and progress, a time to play with the baby and a time to listen to the parents.

Frequency of home visits: - 1 every month from birth to 1 year
- 1 every 2 months from 1 year to 2 years
- 1 every 6 months from 2 to 4 years

From 2 to 4 years old, the referring childcare worker will contact the family by phone between 2 VADs, i.e. at 27 months, 33 months, 39 months and 45 months. This call will allow the childcare worker to speak with the family, to remind them of the possibility of adding VAD at the family's request or to offer them additional VAD if she herself deems it necessary.
Our main objective being to meet the needs of families, the support between 2 and 4 years old will be done according to the identification and expression of the needs expressed by the families, with at least 1 VAD every 6 months and a phone call 3 months after the VAD.

Justification for the modification of the rhythm of VADs between 2 and 4 years of age (substantial modification N°5 to the protocol): following the clinical feedback of the paediatric nurses involved in the follow-up of the families, we considered that the imposed follow-up was too intense. We have therefore opted for the free choice of families to request more VAD if they wish and to replace 1 in 2 VAD with a phone call to still maintain the link with the family.

During the VAD carried out at the child's 4th birthday, a medical intern from the PMI will accompany the referring paediatric nurse in order to carry out a child development test (GED scale: Child Development Evaluation Grid). If a developmental delay is noted, the paediatric nurse or medical intern will suggest an appointment to the family as part of the PMI consultations in the sector in order to be able to initiate a follow-up or referral if necessary.
The development test can also be carried out by the medical intern of the PMI close to the VAD carried out by the paediatric nurse but during a separate VAD, depending on the constraints of each person's agenda and the wishes of the family.
In addition to the child development test, the medical intern of the PMI will carry out an ERTL4 test (Test for the Identification of Language Disorders in 4-year-old children). The initial version of the study protocol already included retrieving data from

the ERTL4 that the childcare workers systematically take as part of the assessments in nursery school. In order to meet the validation deadlines for this test (between 3 years 9 months and 4 years 6 months), we considered it preferable that it be carried out directly at home by the medical intern because the dates of the assessments carried out in the schools vary greatly depending on the childcare nurses and cannot be adapted to the age of each child in the research to meet the validity criteria according to the exact age.
In the event that the ERTL4 test is not taken during the VAD performed for the child's 4 years of age (modified appointment, cancelled appointment, etc.), the ERTL4 test data used for our study will be those recovered during the systematic assessment carried out in the nursery school as initially planned by the study protocol.

Justification for the modification of the addition of a developmental test at 4 years old (substantial modification N°5 to the protocol): it seemed important to us to be able to complete our evaluation of the child's 4 years with a global developmental test. Initially, only the data from the ERTL4 was planned, which is done by the childcare workers as part of the Nursery School Assessments. We did not have the possibility to do a development test 4 years before, as we did not have the necessary material and human resources. Now, we can meet these criteria, which is why we are adding this evaluation, in order to expand the protocol in terms of scientific rigor to meet our research objectives.

- <u>Home visits carried out in pairs by the coordinating psychologist and the family's paediatric referent</u> whose objective is the evaluation of three areas: child development, parenthood and parent-child interaction. These evaluations can be filmed at certain times and these films can be returned to the parents on their request.

Frequency of joint home visits: - 1 to 4 months
- 1 to 24 months

Justification for the modification of 3-month VAD to 4-month VAD (substantial modification N°3 to the protocol): After having carried out the first VAD of assessment at 3 months of the babies, it seemed to us that the data collected at the BLR test were not sufficiently representative of the babies' skills, due to the tool. These clinical elements have been confirmed by data from the literature which report that "the calculation of the developmental quotient is not carried out before 4 months, because, during the first three months, this calculation would not make sense because it would be too high" (Manuel du Brunet-Lezine-Revisé 1997).

➢ <u>*For "control" families*</u>

<u>Home visits carried out by the coordinating psychologist</u> and whose objective is the evaluation of three areas: child development, parenting and parent-child interaction. These evaluations can be filmed at certain times and these films can be returned to the parents on their request.

Frequency of home visits: - 1 to 4 months
- 1 to 24 months

Home visit carried out by a medical intern from the PMI, in order to carry out a child development test (GED scale: Child Development Evaluation Grid). If a developmental delay is noted, the medical intern will propose an appointment to the family as part of the PMI consultations in the sector in order to be able to initiate follow-up or guidance if necessary.

This home visit by a medical intern from the PMI will be carried out only once, at the child's 4th birthday.

In addition to the child development test, the medical intern of the PMI will carry out an ERTL4 test (Test for the Identification of Language Disorders in 4-year-old children). The initial version of the study protocol already provided for the recovery of the ERTL4 data that the childcare workers systematically pass on as part of the assessments in nursery schools. In order to meet the validation deadlines for this test (between 3 years 9 months and 4 years 6 months), we considered it preferable that it be carried out directly at home by the medical intern because the dates of the assessments carried out in the schools vary greatly depending on the childcare nurses and cannot be adapted to the age of each child in the research to meet the validity criteria according to the exact age.
In the event that the ERTL4 test is not taken during the VAD performed for the child's 4 years of age (modified appointment, cancelled appointment, etc.), the ERTL4 test data used for our study will be those recovered during the systematic assessment carried out in the nursery school as initially planned by the study protocol.

Justification for the modification of the addition of a developmental test at 4 years old (substantial modification N°5 to the protocol): it seemed important to us to be able to complete our evaluation of the child's 4 years with a global developmental test. Initially, only the data from the ERTL4 was planned, which is done by the childcare workers as part of the Nursery School Assessments. We did not have the possibility to do a development test 4 years before, as we did not have the necessary material and human resources. Now, we can meet these criteria, which is why we are adding this evaluation, in order to expand the protocol in terms of scientific rigor to meet our research objectives.

- *For "control" and "intervention" families*

  - 10 families from each group (intervention and control), drawn at random, will benefit from a visit (at home or in the maternity ward according to their convenience), by a psychologist from the Psychotherapy Center of Nancy who carried out the INTERREG research (Elise Fidry) in order to evaluate the baby's development and highlight his skills to the parents. This evaluation will be carried out blindly by the psychologist who does not know the group to which the family belongs (witness or intervention).

    Rationale for adding the Brazelton test: Given the first VADs performed and the data in the literature (see justification for modifying the first VAD), we opted for a 4-month VAD. However, by moving the VAD of evaluation, an additional VAD to the intervention group is carried out by the paediatric nurse and there may already be effects on the child's development. It is therefore important to have

an initial evaluation upstream in order to check the homogeneity of the groups in terms of the baby's development, which can be achieved by taking the Brazelton test, a test that requires special training of the evaluator.
Initially we did not offer this test due to a lack of trained and available professionals, which is now possible because a trained psychologist has been able to free up time to be able to carry out these 20 Brazeltons.

- At least 10 GED evaluations in each group will be filmed in order to perform a double rating of this test: one scored by PMI's internal during the VAD and one blinded by one of the other PMI interns trained in the GED evaluation for the PERL study.
  Indeed, it is planned that a maximum of 3 interns carry out the GED test every 4 years of age of the children, except unforeseen circumstances requiring the intervention of an additional intern.
  The interns carrying out the GEDs will all be trained in the tool before taking the test with the children of the research.
  The double rating (thanks to the filmed interviews) will make it possible to ensure the reproducibility of the ratings between the interns, within each group (control and intervention) and between the groups and to be able to take into account any bias identified during the analyses.

2.5 Assessment Tools

2.5.1 Sociodemographic and family data

Different socio-demographic and family data will be collected in the groups "intervention" and "controls" (age, level of education, professional activity and family situation, of both parents). We will also collect about the child: sex, sibling rank, birth weight and gestational age. We will also note: the type of childbirth, parity, the type of childcare, schooling and the age of first schooling, the desire to have children, the mode of feeding (breastfeeding or bottle-feeding), and the father's investment in research, whether or not there is a family support measure.

Finally, we will rate the presence of two factors: the traumatic experience of birth and the pathology of the deficiency based on the criteria defined by Lamour in 2015, which are 4 in number: health and social deficiencies, lack of organization in daily life/discontinuity, history of parents marked by deficiency and mistreatment and frequency of a serious psychopathology in the parent (but often unrecognized and untreated).

2.5.2 Evaluation longitudinale

A longitudinal evaluation will be carried out in both groups in order to establish an evolutionary profile of the different dimensions studied:
- the development of the baby;
- l'interaction ;
- the experience of parenthood;
- the baby's relational investment/withdrawal;
- postpartum depression;

- the experience of the accompaniment;
- Maternal psychiatric symptomatology

2.5.3 Evaluation comparative

The effects of the support will be evaluated by comparison with a control population, which will not have benefited from home support by the childcare workers but only from the 3 evaluation meetings at 4 months, 24 months and 4 years.

The comparative evaluation at 4 and 24 months in the intervention and control groups will focus on:

- the child's psychomotor and language development;
- the experience of parenthood;
- the child's social-emotional development;
- the baby's relational investment/withdrawal;
- postpartum depression;
- Maternal psychiatric symptomatology
- language delays at 4 years old;
- the need to seek care at 4 years of age.

The comparative assessment at 4 years of age in the intervention and control groups will focus on the global assessment of the child's development.

2.5.4 Methodological plan

**Age:**	**Birth**	**4 months**	**24 months**	**4 years**
**POPULATION CONCERNED:**	10 families intervention 10 control families	INTERVENTION AND WITNESSES	INTERVENTION AND WITNESSES	INTERVENTION AND WITNESSES
Child Development	Brazelton	Brunet Lézine Revised ADBB	Brunet Lézine Revised ADBB BITSEA	ERTL4 GED
Parenting experienc e Experienc e of intervention by families		E PR	E PR	
Early interactio ns		PIM13 (video analysis)	PIM13 (video analysis)	
Therapeuti c alliance		WAI Only to the Intervention group	WAI Only in the Intervention group	
Symptom assessment maternal psychiatric		SCL-90 R EPDS	SCL-90 R BDI	
Social Affluence		FAS	FAS	

- Brazelton test: Scale for assessing the development of the baby from birth to 2 months. This tool evaluates the emotional and behavioral characteristics of the newborn through a clinical examination involving the parents.
- Brunet-Lézine-Revised Test (BLR): assessment of psychomotor development from 0 to 30 months;
- ERP: Interview on Parental Representations;
- ERTL4: Test for the Identification of Language Delays at 4 years of age, carried out systematically by the Maternal and Child Protection teams and during the VAD of the child's 4 years of age;
- GED: Evaluation grid for the development of children from 0 to 6 years old. This grid has been validated in Canada and a French validation study is underway. This tool has the advantage of being correlated with the results obtained on the BLR (Child Development Assessment Test taken at 4 and 24 months). In addition, this test involves the parents during the test.
- ADBB: filmed evaluation of the relational withdrawal of young children from 0 to 24 months (video);
- EPDS: Edinburgh Postnatal Depression Scale. Postpartum Depression Rating Scale;
- BDI: Beck's Depression Inventory. Scale that assesses the severity of depression in adults;
- BITSEA: Brief Infant-toddler Social and Emotional Assement. Early detection scale for behavioural disorders and developmental delays between 1 and three years.
- PIM 13: Parent-infant, early Interactions Measure 13 items: tool developed by Mrs. Sophie BUCHHEIT and Prof. Fabienne LIGIER. This grid includes items to assess the quality of early interactions and maternal sensitivity. Attachment is in the background of this grid.
- FAS: Social Affluence Assessment Scale to Determine the Socio-Economic Level of Families
- SCL-90 R: Symptom Check List: Comprehensive Psychiatric Symptom Self-Assessment Questionnaire
- WAI: Working Alliance Inventory (WAI) (Horvath, 1994) [27]: this is a therapeutic alliance scale widely used in Anglo-Saxon studies, which has been translated into French and adapted to the context of early parent-young child consultations. The scale includes 12 questions exploring four dimensions of allyship: therapist-parent relationships (3 questions), therapist-child alliance (3 questions), the "positive" dimension of goals and tasks (3 questions), and the "negative" dimension of goals and tasks (3 questions whose answer is reversed from the others). The answers are given on a 7-point scale, 3 of which are defined (1= strongly disagree; 4=moderately agree, 7=strongly agree). In this study, the scales will be slightly modified (replacement of "consultations" by "encounters", and of "consultant" by "childcare worker") in order to adapt to the context of PMI's work. The childcare worker who will follow the family and the mother will fill in the evaluation scales of the alliance.

The majority of the tests used have been validated in French, the GED has been validated in Canada and a French validation study is underway and they have good psychometric qualities. Only the Interview on Parental Representations and the PIM 13: Parent-infant, early Interactions Measure 13 items have not been scientifically validated. However, the ERP is a tool that has been specifically designed for the first action research (INTERREG). To develop this instrument, we have based ourselves on the contributions of Stern et al. (1989) on

the "R" interview and the PDI, which are methods for evaluating maternal representations. The PIM 13 was built by members of our scientific and medical team (Mrs. Sophie BUCHHEIT, Prof. Fabienne LIGIER). We integrated items to assess maternal sensitivity and the quality of early interactions. Attachment is in the background of this grid. For the construction of this observation grid, we were inspired by several validated tests (French and/or international): CIB (Coding Interactive Behavior), CARE-INDEX, MBQS (Maternal Behavior Q-Sort), AQS (Attachment Q-Sort), DMC (Dyadic Mutuality Code), GRS (Global Rating Scale), GEDAN (Adult-Infant Dialogue Evaluation Grid). We also consulted Steinhauer's guide, the ADBB (Distress Alarm-Baby) and the PIPE (Pediatric Infant Parent Exam). The creation of this tool was recommended to us by Professor Antoine Guedeney, a professor of psychiatry specializing in our field of research.

2.5.5 Analysis of processes and mechanisms

It should be noted that the longitudinal and comparative evaluation of this research is associated with an analysis of the processes and mechanisms that will be carried out by the team of the APEMAC laboratory (School of Public Health, Laboratory of Chronic Diseases, Perceived Health and Adaptation Processes). Epidemiological and psychological approaches). More specifically, it will involve:

1) define and validate the intervention theory (what do the levers used produce and how, what are the mechanisms linking the intervention to its results),
2) describe the location and processes involved,
3) Analyze the effect of context, including social context, on processes and outcomes. This analysis will be the subject of a specific protocol of the Nancy School of Public Health.

2.6 How data is processed

2.6.1 Descriptive analysis

A descriptive analysis will be done to:
- Socio-demographic characteristics
- Vulnerability factors

2.6.2 Statistical analysis

An intermediate statistical and qualitative analysis will be carried out two years after the start of the study to confirm that there are no deleterious elements for the participants. This statistical analysis will be carried out by the research coordinator as well as by Professor Fabienne LIGIER (Doctor of Public Health).

The statistical tests used will be as follows:

- The student's t test will be used to compare the quantitative variables:
    * To compare the homogeneity of the two groups (intervention and controls) with regard to the variables age of parents, birth weight of the child, gestational age.
    * compare the results obtained at Brunet-Lezine, ERTL4, BDI, BITSEA, and ADBB between the two groups.
    * To analyze the impact of the tone of parental representations on the scores obtained on the different scales.

- The Chi-2 (χ^2) test will be used to:
  * compare the homogeneity of the two groups for the qualitative variables concerning the sex of the child, the rank in the siblings, the level of education of the parents, the parents' professional activity and the family structure For the families concerned, there will also be a comparison of the scores of the Brazelton test.
  * observe whether a difference appears in the tone of the Parental Representations at the interview (experience of parenthood) between the two groups.

- Spearman's Rho correlation coefficient will be used for correlations between scores obtained on the different scales (Brunet-Lézine, ADBB, EPDS, BDI, BITSEA, WAI, FAS, SCL-90 R).
All conditions of use of the tests will be checked before they are used.

3. METHODOLOGICAL PRECAUTIONS AND ETHICAL GUARANTEES

3.1 Methodological precautions

3.1.1 Tests

All the methodological tools meet different characteristics:
- they must report comparative and transmissible data;
- they must not be too intrusive, the handover must not be too burdensome, neither for the worker nor for the parent (so as not to "go against" the objective of prevention, and so that the program is easily reproducible);
- the observation grids must be as descriptive as possible, to enrich the quality of the data and to differentiate between evaluation time and analysis time;
- The different tools go in the same direction and contribute to our prevention objectives (we seek to highlight resources, skills, encourage interaction, etc.).
- the grids and questionnaires used are validated in French (or English) and for those that are not, they have been the subject of French and/or international publications, with the exception of the PIM13 grid, which has nevertheless been built on the basis of validated tests (see section 2.5.4).

3.1.2 Anonymity

When families are considered part of the study, we will proceed with anonymization. To do this, a number will be assigned to the inclusion of each family: 1 for intervention 1 and T1 for Control 1. The nominative data will not be kept in the same place and on the same medium as the anonymised data and the list of assigned numbers, in order to guarantee anonymity and so that no aggregation can be made.

3.1.3 Data collection

All the data collected during the 4 years of research will be collected on a paper observation sheet and will constitute the source documents associated with the results of the evaluations. The data required for the research will be stored on a laptop and an external hard drive (anonymized); only Mrs. BUCHHEIT will have access to it. The computer will not be accessible to a third party, locked by a password and a card (Secure Professional Card) and will be locked in a cupboard at the Medical Centre

Psychological for children and adolescents in Vandoeuvre, Mrs. Buchheit's administrative residence.
The code assigned at the time of inclusion of the family will be the only one to appear on the data collected.

The regulations of the CNIL will be applied for the identification of participants. We will not collect dates of birth, however, if it were to be mentioned, it would be limited to the months and year of birth (the day being too identifying).

All the data collected will be kept and destroyed 15 years after the end of the research.

3.2 Ethical safeguards

3.2.1 Scientific committee

This research is supported by a multidisciplinary scientific committee that meets every two months to take stock of the progress of the research and to ensure that all ethical and methodological criteria are respected.
This working group is made up of:

- S. BUCHHEIT, Clinical Psychologist, Project Coordinator, Nancy Psychotherapy Center
- Prof. B. KABUTH, Professor of Child Psychiatry, University of Lorraine, Head of the Child Psychiatry Unit of the Psychotherapy Center of Nancy
- Prof. F. LIGIER, child psychiatrist, doctor of public health, Nancy Psychotherapy Center.

As well as one or more representatives of the following structures: ARS Grand Est, Departmental Council, PMI, CAF du Lunévillois, Department of Prevention and Promotion in Mental Health of the Psychotherapy Center of Nancy; as well as child psychiatrists, psychologists and health executives from the Nancy Psychotherapy Center and health executives from the Departmental Council.

Representatives of the Nancy School of Public Health will provide methodological advice on the project as a whole and will be present at the meetings of the Scientific Committee throughout the evaluation of the processes and mechanisms.

3.2.2 Documents

Consent
After the families have given their consent to participate in this research, they will have to sign the consent form. A copy of this consent will be given to them as well as a copy of the information note.
The consent form must be signed by both parents with parental authority, and failing that, by the parent with exclusive parental authority in the event that the father has not recognized the child or if the latter is absent at the time of the presentation of the study (as provided for in Article L1122-2 of the Public Health Code, *"the other holder of the exercise of parental authority may not give his authorization within a period compatible with the methodological requirements specific to the conduct of the research, with regard to its purposes")*). Nevertheless, the research coordinator remains available for any request for additional information from the father.

Information note

The briefing note will be read with the families and explained before a copy is provided to them.

Image rights

A document of access to the image will also have to be signed by the parents to obtain their consent and authorize us to use the videos we will make for the rating of the evaluations, as described in the protocol. A copy will also be given to them.
This document must, like the informed consent, be signed by both parents with parental authority over the child.

Data protection:

- CNIL

This study does not fall within the scope of the CNIL's MR001 reference methodology due to the production of identifying videos among the families recruited for the study.
Authorization from the CNIL is required before the study is implemented. The necessary steps have been taken by the sponsor of this study.

- GDPR

In accordance with the General Data Protection Regulation (GDPR), the persons participating in this research will be informed, via the information notice and the consent form, of the following rights:

- the identity and contact details of the data controller, i.e. the sponsor
- the contact details of the data protection officer
- the legal basis for the data processing
- the nature and purpose of the data collected in the context of the research as well as the period of storage of this data
- the possibility of stopping the study at any time and the retention by the promoter of the information collected (unless otherwise specified by the person concerned).
- their rights of access, rectification, opposition, limitation and deletion of data collected in the context of research. These rights may be exercised at any time during the research by making a request to the sponsor's Data Protection Officer
- the possibility, in the event of a problem/disagreement, to file a complaint with the CNIL.

The promoter undertakes to respond to any request for access to the data within a maximum period of 1 month. This period may be extended by 2 months depending on the complexity and number of requests. In addition, only staff authorised by the sponsor (investigators, ARC, TEC) and representatives of the health authorities will be able to have access to this information.

3.2.3 Vigilance

This study is part of the Research Involving the Human Person, category 2 (minimal risks and constraints) according to Law No. 2012-300 of March 5, 2012 (Jardé Law).

As such, no specific vigilance monitoring circuit is required for this study on the part of the sponsor.

Only adverse events noted by the investigator or reported by families during their participation in the study will be recorded in the study case report.

If these adverse effects are subject to health vigilance (pharmacovigilance for adverse drug effects, materiovigilance for an effect related to a medical device,...), they will be transmitted by the investigator according to the usual reporting procedure to the health vigilance concerned, without notifying the sponsor.

4. BENEFITS/RISKS OF RESEARCH

4.1 Benefits

We expect to measure a beneficial effect of a joint childcare intervention (from the Maternal and Child Protection) and psychologist (from child psychiatry) on the child's development and the experience of parenthood.
We expect a significant reduction in developmental and language delays and a better experience of parenthood for the families benefiting from the support.

4.2 Risks

There is no risk identified in this action research.
It is important to specify that the "control" and "intervention" cohorts will be able to benefit from a classic PMI and CMP follow-up if they wish.
This study does not penalize the "control" cohort in any way in the usual follow-ups that could have and can be set up in the perinatal period.
In addition, in the event that difficulties are detected during the evaluation visits of the control population, we will inform the parents of our concerns and they will be directed to a care structure adapted to the difficulties identified. This configuration will not hinder their participation in this research.

4.3 Allowances

There is no financial compensation provided for this research. There are no financial costs to the families involved in this research.

There is no prohibition on participating in another research project at the same time while participating in the SERP action research.
There is no deferral period during which participation in other research is prohibited.

5. RESEARCH FUNDING

5.1 Funding of posts

This research has obtained multi-institutional funding:

- the departmental council makes sector childcare workers available to participate in research (1.60 FTE);
- the Nancy Psychotherapy Centre finances the position of the psychologist who coordinates and evaluates the research as well as the position of the psychologist who will do the supervision (1 FTE);
- the Regional Health Agency provides financial support for the position of psychologist;
- the Family Allowances Fund provides financial support for the position of psychologist.

5.2 Budget

The entire budget to finance the PERL action research has been allocated. This budget includes:
- the 10 game kits that will be available to childcare workers for home visits;
- all the tests used by the psychologist during the evaluations;
- the computer and external hard drive used to process the data collected;
- reimbursement of the psychologist's transport costs;
- insurance.

6. DATA COLLECTED AND SOURCE DOCUMENTS

Description of the variables collected
Data from the study will be collected as described in section 3.1.3. The information collected in this study is described in section 2.5.

Identifying source data
The source documents will consist of all the information and test results used in the framework of this study, contained in a file specific to the research and centralized at the CMP children and adolescents in Vandoeuvre.
The following information will be included in the file of each family participating in the study:

- title of the study,
- date of information and inclusion of the patient in the study (signature of consent),
- the patient's various visits as part of the protocol,
- the occurrence of Adverse Events (Serious).

The coordinating investigator undertakes to give direct access to all these documents to the persons mandated by the sponsor, as well as to representatives of the health authorities.

7. QUALITY CONTROL AND ASSURANCE

Quality control will be carried out by a Clinical Research Associate (CRA) mandated by the sponsor according to the level of monitoring defined beforehand.

8. ETHICAL AND REGULATORY CONSIDERATIONS

This is a study falling within the framework of Law 2012-300 (known as the Jardé Law) of 5 March 2012, category 2.

Compliance with the regulatory provisions in force
The research will be conducted in accordance with the protocol, good practices and the legislative and regulatory provisions in force
The protocol is in line with the ethical principles established by the Declaration of Helsinki

Protection of people
Before starting the inclusions, the protocol will be submitted to the opinion of the Committee for the Protection of Persons North-West IV in Lille.
It will be sent to the ANSM for information.
The procedures for obtaining consent are described in paragraph 3.2.2

Insurance
The study sponsor has taken out insurance covering its civil liability and that of any intervener, regardless of the nature of the relationship between the interveners and the sponsor.

Registration in the national VRB file (BioMedical Research Volunteers)
There is no prohibition on participating in another research simultaneously while participating in this study.
There is no deferral period during which participation in other research is prohibited.
No compensation to participants is required in this study. As a result, this study does not require the registration of participants in the VRB file.
