# Supplementary material for: Development and validation of the MIPPE: A novel dyadic assessment tool for early parent-child interactions in clinical practice
Source: PLoS One. 2026 Apr 24;21(4):e0347521. doi: 10.1371/journal.pone.0347521 (PMC13108784; doi:10.1371/journal.pone.0347521)
Supplement: S7 File — (PDF) [file pone.0347521.s007.pdf]

Evaluer les effets d'un accompagnement préventif à domicile sur le développement du jeune enfant
PERL : Petite Enfance, Recherche-action en Lorraine

Centre Psychothérapique de Nancy
1, rue du Docteur Archambault
BP11010
54521 LAXOU Cedex
Téléphone : 03.83.92.50.50

MDD Lunéville
Service de PMI
28, rue de la République
54300 LUNEVILLE
Téléphone : 03.83.74.44.24

Coordinatrice du projet : Sophie BUCHHEIT
Courriel : sophie.buchheit@cpn-laxou.com

PROTOCOLE DE LA RECHERCHE PERL
incluant la modification substantielle N°7

Titre
Evaluer les effets d'un accompagnement préventif à domicile sur le développement du jeune enfant
PERL : Petite Enfance : Recherche action en Lorraine

Promoteur
Centre Psychothérapique de Nancy, 1 rue du Docteur Archambault, BP11010 – 54 521 Laxou Cedex

Investigateur coordinateur
Sophie BUCHHEIT

Version du protocole
Version 9 du 23/06/2023 incluant la modification substantielle N°7, ayant reçu un avis favorable du CPP Nord Ouest IV le 24/08/2023.

Avis favorable initial du CPP Nord Ouest IV le 14/11/2017
Avis favorable du CPP Nord Ouest IV pour la modification substantielle N°1 le 25/01/2018
Avis favorable du CPP Nord Ouest IV pour la modification substantielle N°2 le 20/12/2018
Avis favorable du CPP Nord Ouest IV pour la modification substantielle N°3 le 23/04/2019
Avis favorable du CPP Nord Ouest IV pour la modification substantielle N°4 le 26/09/2019
Avis favorable du CPP Nord Ouest IV pour la modification substantielle N°5 le 27/05/2021
Avis favorable du CPP Nord Ouest IV pour la modification substantielle N°6 le 18/08/2022

Type d'étude
Recherche interventionnelle à risques et contraintes minimes, mentionnée au 2° de l'article L.1121-1 du Code de la santé publique.

Numéro ID RCB
2017-A00896-47

1. INTRODUCTION

1.1 Justification/contexte

Pour lutter contre les inégalités sociales de santé (qui est une des priorités de la politique de santé en France), la petite enfance est une période clef. Dans ce cadre l'accompagnement à la parentalité est reconnu comme un levier potentiellement efficace (in *World Health Organization. Closing the gap in a generation: health equity through action on the social determinants of health. Final Report [Internet]. Geneva: Commission on Social Determinants of Health; 2008 p. 256. ; Guyer B, Ma S, Grason H, Frick KD, Perry DF, Sharkey A, et al. Early Childhood Health Promotion and Its Life Course Health Consequences. Acad Pediatr. 2009 May; 9(3):142–149.e71. ; Barlow J, Smailagic N, Huband N, Roloff V, Bennett C. Group-based parent training programmes for improving parental psychosocial health. In: Cochrane Database of Systematic Reviews [Internet]. John Wiley & Sons, Ltd; 2014 [cited 2016 Jan 8]. ; Barlow J, Smailagic N, Bennett C, Huband N, Jones H, Coren E. Individual and group based parenting programmes for improving psychosocial outcomes for teenage parents and their children. In: Cochrane Database of Systematic Reviews [Internet]. John Wiley & Sons, Ltd; 2011 [cited 2016 Feb 24]. ; Shonkoff JP, Boyce WT, McEwen BS. Neuroscience, Molecular Biology, and the Childhood Roots of Health Disparities: Building a New Framework for Health Promotion and Disease Prevention. JAMA. 2009 Jun 3;301(21):2252. ; Postnatal parental education for optimizing infant general health and parent-infant relationships. Cochrane Database Syst Rev. 2013 Nov*) et les actions d'accompagnement à la parentalité sont promues.

Plus spécifiquement sur le territoire du Lunévillois, il est observé chaque année une fréquence importante de retards de langage chez les enfants de 4 ans au test ERTL4, réalisé de façon systématique par la PMI à l'école maternelle. Ces chiffres révèlent un contexte de fragilité et de précarité des familles et nous incitent à proposer des accompagnements le plus précocement possible.

Une recherche-action (projet Interreg) proposée aux enfants nés en 2010 et à leurs parents sur les secteurs de Blâmont, Cirey-Sur-Vezouze et Badonviller a montré que des visites à domicile réalisées par une psychologue (in *Prendre soin du bébé et de sa famille : une expérience de recherche-action en périnatalité / Fidry Elise ; Claudon Philippe ; Saad Saint-gilles Stéphanie ; Sibertin Blanc Daniel. Neuropsychiatrie de l'enfance et de l'adolescence, 2014, n° 3 ; Des premiers regards aux premiers mots : une expérience lorraine de prévention en périnatilité / Fidry Elise ; Sibertin Blanc Daniel ; Claudon Philippe. -Paris : L'Harmattan, 2014. - 65-83*) ont permis de **réduire significativement les retards de développement et les retards de langage**, avec :

1) 39 % de retards au test de développement à 24 mois dans la cohorte intervention contre 74% de retards dans la cohorte témoin ;
2) 21% de retards au test de langage à 4 ans dans la cohorte intervention contre 31% dans la cohorte témoins ;
3) nécessité de soins (orthophonique ou psychologique) pour 23% des enfants de la cohorte intervention contre 49% des enfants témoins.

L'objectif du projet PERL est d'utiliser ce modèle d'accompagnement à domicile et de l'adapter pour le proposer sur un secteur élargi, tout en l'intégrant dans les pratiques existantes, dans le cadre d'un partenariat entre la Protection Maternelle et Infantile et la pédopsychiatrie. Un dispositif innovant en matière de prévention pour soutenir les interactions et le

développement de l'enfant sera proposé à une soixantaine de familles, recrutées aléatoirement en population générale. Si la famille accepte de participer, elle bénéficiera alors de visites à domicile régulières par une puéricultrice accompagnée sur certains temps d'une psychologue, pendant une durée de 4 ans à partir de la naissance de l'enfant. Cet accompagnement à domicile, centré sur l'écoute et l'observation, s'articule autour de trois axes : le développement du bébé, la parentalité, l'interaction parents-enfant. Conjointement à la mise en œuvre pratique de ce dispositif, nous nous proposons de l'évaluer selon un plan méthodologique préétabli.

Cette recherche-action s'inscrit dans une dynamique partenariale forte et elle est portée par plusieurs institutions : Conseil Départemental de Meurthe et Moselle, Centre Psychothérapique de Nancy, Agence Régionale de Santé Grand-Est, Caisse des Allocations Familiales de Meurthe et Moselle.

1.2 Objectif principal de la recherche PERL

Evaluer les effets de l'accompagnement préventif à domicile par une professionnelle de la petite enfance sur la prévalence des retards de langage et de développement des enfants par rapport à la prise en charge habituelle.

1.3 Objectifs secondaires de la recherche PERL

1- Evaluer de façon longitudinale : le développement de l'enfant jusqu'à ses 4 ans, la qualité des interactions parents-enfant et le vécu de la parentalité.
2- Evaluer de façon longitudinale le vécu de l'accompagnement à domicile.

2. ASPECTS METHODOLOGIQUES

Les cohortes, seront soumises à une évaluation longitudinale et comparative intergroupe.

2.1 Population

Il est prévu d'inclure :
- 64 familles pour le groupe « intervention » ;
- 64 familles pour le groupe « témoins ».

Un calcul de puissance a été réalisé : Si l'on part sur un cas pour un témoin, avec une puissance à 80% un risque alpha de 5%, une prévalence de retard de développement à 50% chez les témoins et 25% chez les patients bénéficiant du dispositif, nous obtenons un résultat à : 58 personnes groupe intervention et 58 personnes groupe témoins. En tenant compte d'une perte de 10 % de sujets au cours de la recherche, nous obtenons 64 sujets à recruter groupe intervention et 64 sujets groupe témoins pour cette recherche-action.
De plus, le chiffre de 64 a également été estimé pour répondre aux contraintes de contexte pratique des puéricultrices, qui conditionne la limite de sujets pouvant être pris en charge. Les puéricultrices poursuivent leur activité habituelle en PMI, à laquelle se rajoutent les VAD (visites à domicile) pour la recherche PERL. Il nous a donc paru pertinent, au vu de la poursuite de leur activité professionnelle, et après discussion avec l'équipe du Conseil Départemental (service de PMI), d'attribuer 8 familles par puéricultrice. Initialement, nous avions convenu d'un suivi de 8 familles par puéricultrice, après échange avec le médecin de territoire (PMI). Suite à la mise en place des premières VAD réalisées par les puéricultrices, celles-ci nous ont

informés que le nombre maximum de familles avec lesquelles elles pourraient mettre en place un suivi durant 4 ans était de 4 par puéricultrice. Dans le but d'obtenir nos 64 familles pour le « groupe intervention », nous avons élargi le secteur de recrutement de l'étude (du Pays Lunévillois à la Meurthe et Moselle sud) afin de pouvoir impliquer davantage de puéricultrices (6 puéricultrices supplémentaires) et disperser la charge de travail.

Cette recherche sera proposée aux parents résidant dans le secteur de Meurthe et Moselle Sud et ayant donné naissance à un enfant au cours des années 2018 à 2020. Les critères d'inclusion sont donc au nombre de deux :

- naissance de l'enfant au cours des années 2018 à 2020 ;
- lieu de résidence (Meurthe et Moselle Sud).

Les critères de non inclusion sont au nombre de deux :

- Les familles ne parlant pas français, la barrière de la langue ne permettant pas la compréhension claire des items de certains tests et rendant difficile l'échange avec la puéricultrice.
- Les gens du voyage non sédentarisés, qui ne pourront pas bénéficier des VAD de la naissance aux 4 ans ou des VAD aux 4 et 24 mois

Pour cette recherche nous avons fait le choix de travailler en population générale et de ne pas recruter les familles en fonction de critères de vulnérabilité. Cela nous permettra d'éviter la stigmatisation des familles et de nous concentrer sur leurs aptitudes.

Par ailleurs, le recrutement initial pour l'étude PERL était limité au secteur du Lunévillois. Ce secteur avait été retenu afin d'être au plus près du secteur géographique de la première recherche INTERREG. La demande initiale de mise en place de la recherche PERL a, en premier lieu, été une démarche professionnelle du médecin de territoire du Pays Lunévillois (PMI). Nous avons fait le choix d'étendre ce périmètre de recrutement et de suivi des familles au secteur de Meurthe et Moselle Sud car le manque de moyens humains (professionnelles de PMI) sur le territoire du Lunévillois entraine une charge de travail trop importante pour les puéricultrices qui suivent les familles inclues dans le « groupe intervention ». Afin que la période d'inclusion reste dans des délais raisonnables et cohérents avec nos objectifs scientifiques, il nous a paru nécessaire d'étendre le périmètre de recrutement afin que les puéricultrices d'autres secteurs puissent intervenir dans le cadre de l'étude PERL (modification substantielle N°4 au protocole). L'extension à la Meurthe et Moselle Sud permet de conserver un territoire équivalent en termes de caractéristiques de la population (résultats au test de développement du langage et profil de population) et de ne pas créer de biais dans l'analyse ultérieure des données recueillies.

2.2 Modalités de recrutement des familles

2.2.1 Courrier systématique

Les familles ayant déclaré leur grossesse et dont la date de terme est prévue entre septembre 2018 et décembre 2020 recevront toutes un courrier les informant que la recherche PERL a débuté en 2018 et qu'elles sont susceptibles d'être contactées pour leur proposer d'y participer.

Suite à ce courrier, les familles peuvent nous contacter pour s'opposer à cette démarche sur simple appel téléphonique, mail ou courrier. Si elles n'émettent aucun refus, elles feront partie de la liste avec laquelle nous procéderons au recrutement aléatoire pour déterminer les familles faisant parties de la cohorte « témoins » et celles faisant partie de la cohorte

« intervention ». Une fois les familles contactées, l'ensemble des données sera anonymisé de façon systématique.

La cohorte « témoins » sera donc sélectionnée sur le même territoire et d'après un recrutement aléatoire identique à la cohorte « intervention ».

2.2.2 Recrutement aléatoire

Toutes les familles participant à l'étude seront recrutées sur la base du volontariat. Elles seront sélectionnées par un recrutement aléatoire (randomisation de Zelen) réalisé tous les mois afin d'atteindre le nombre d'inclusions attendu (64 interventions et 64 témoins). Il a été convenu, en accord avec le comité scientifique de la recherche, de sélectionner les huit premières familles tirées au sort et acceptant de participer à la recherche de chaque mois jusqu'à atteindre 8 inclusions chez les personnes bénéficiant de l'intervention (une famille par mois par puéricultrices) et 8 inclusions chez les témoins par mois.

2.2.3 Première information par courrier

Les familles recrutées de façon aléatoire seront informées qu'elles sont concernées par la recherche-action PERL aux environs du 8ème mois de grossesse afin de ne pas inclure les accouchements prématurés (qui constitueraient un biais dans l'interprétation des données quantitatives recueillies au cours de certains tests de développement). Un courrier leur sera adressé à leur domicile, accompagné d'une plaquette de présentation de la recherche-action. Un rendez-vous avec la psychologue coordinatrice leur sera proposé à leur domicile ou dans une structure de la petite enfance, à leur convenance. Cette rencontre permettra de transmettre aux parents les informations concernant la recherche-action PERL sans que cela ne les engage à y participer.

2.2.4 Informations données lors de la première rencontre

Si cette première rencontre est acceptée par les parents, celle-ci aura lieu au cours du huitième mois de grossesse, afin de leur présenter cette recherche. Une information détaillée sera faite par la coordinatrice concernant les modalités pratiques (nombre et rythme des visites à domicile, contenu des échanges, personnes présentes, durée,…), leurs droits (d'arrêt de la recherche, de récupération des données les concernant,…), le traitement des données (anonymat, conclusions globales et non individuelles). Cet échange aura comme support une note d'information qui leur sera ensuite remise, accompagnée par un formulaire de consentement. Ils disposeront ensuite d'un délai de réflexion, afin de décider s'ils souhaitent faire partie de la recherche-action PERL.
S'ils l'acceptent, le formulaire de consentement et la note d'information seront signés, et ils seront considérés comme étant inclus dans la recherche.

2.3 Modèle d'accompagnement préventif

Les puéricultrices et la psychologue coordinatrice ont bénéficié d'un temps de formation de 5 jours (35 heures) avant de débuter le projet autour de l'observation à domicile à visée préventive. Cette formation articulait pratique et théorie pour préparer l'action de terrain. Elle a été délivrée par Elise Fidry, la psychologue chargée de la recherche précédente et par le Dr Stéphanie Saad Saint-Gilles. Cette formation s'étant achevée un an avant le début de la mise en

place de l'action, et étant peu reproductible aux futures puéricultrices qui intègreraient le dispositif, nous avons convenu de réorganiser les modalités de formation. Désormais, les puéricultrices participant au dispositif PERL bénéficieront tous les semestres (à réévaluer en fonction des besoins des puéricultrices selon leur demande) de journées de travail et d'échanges et d'une formation continue à raison d'une heure par mois, après les supervisions (cf. plus bas). Les futures puéricultrices auront un entretien avec la psychologue coordinatrice du projet afin de leur expliciter les modalités de l'étude et de leur présenter le modèle et protocole concernant les Visites A Domicile.

Le modèle d'accompagnement préventif prévoit que les visites à domicile s'articulent autour de trois temps : l'observation conjointe du bébé avec ses parents, l'entretien avec les parents et l'interaction, en particulier au travers du jeu.

L'accompagnement repose sur trois grands axes :
- Le soutien à la parentalité : l'écoute active et attentive du professionnel a pour objectif de favoriser l'émergence de représentations parentales imaginaires, fantasmatiques et narcissiques par rapport au bébé. La parentalité est à considérer comme un processus maturatif dont la temporalité est singulière pour chaque individu. L'accès à la parentalité implique un ensemble de remaniements identitaires et relationnels qui pourront être soutenus dans le cadre des rencontres.
- L'observation du bébé avec ses parents : les temps d'observation du bébé et de ses progrès permettent au parent de s'identifier à cette « fonction observante » et ainsi de favoriser le développement de leur tout-petit.
- Favoriser l'interaction parent-bébé : comme la théorie de l'attachement a pu le démontrer, à la naissance, le bébé est un être social pour qui l'échange interactif auprès d'une figure sécurisante représente un besoin primaire. Dans l'accompagnement, il paraît important de s'intéresser à la construction progressive des liens d'attachement et d'inviter les parents à entrer en relation avec le bébé dans les soins ou par le jeu. Le professionnel peut également renforcer la sécurité du lien en offrant, grâce au cadre de la recherche-action, une régularité et un attachement sécurisant aux parents.

L'accompagnement s'inspire du modèle d'observation à domicile de Bick (1963) qui insiste sur les aspects essentiels de la position de l'observateur : « l'écoute », «la réceptivité», « l'attention consciente et inconsciente ». Il s'appuie également sur les apports du programme de santé mentale infantile de Fraiberg (1989), qui valorise l'observation attentive, l'écoute empathique, la relation de soin nourrissante avec les parents et l'intérêt pour les expériences passées. Le professionnel est présent au bébé et à ses parents dans la continuité et vient assurer une contenance, telle qu'elle a pu être décrite par Bion (1962). Il est attentif au développement du bébé, aux interactions précoces et au vécu de la parentalité.

L'équipe des puéricultrices bénéficiera de deux heures de supervision mensuelle réalisée par Sophie BUCHHEIT, psychologue coordinatrice de la recherche. Ces temps auront pour objectif d'analyser et d'élaborer les observations faites à domicile. Les temps de supervision seront modulables en fonction des conditions de travail des puéricultrices, de l'évolution des conditions sanitaires auxquelles nous devons faire face et nous adapter. Dans le cadre d'une recherche, l'essentiel est de pouvoir s'adapter au terrain, aux professionnelles de terrain, tout en maintenant le cadre des supervisions comme obligatoires dans le cadre du dispositif. Les puéricultrices peuvent également avoir accès à des temps de supervision individuelle une fois par semaine, assurés par Sophie BUCHHEIT. Il est également précisé aux puéricultrices qu'elles peuvent joindre la psychologue coordinatrice sur son téléphone personnel à n'importe quel moment afin d'échanger sur le suivi des familles rencontrées. Ces

deux heures de supervision seront suivies d'un échange centré sur les processus à l'œuvre au cours des rencontres et les apports théorico-cliniques que pourra apporter S. BUCHHEIT (qui rédige une thèse de Doctorat de psychologie portant sur cette recherche). De cette manière, la formation des puéricultrices se poursuivra en parallèle des VAD, et les thématiques abordées pourront être agrémentées de cas clinique concrets concernant le suivi des familles PERL.

2.4 Rythme des visites à domicile

Dans le cadre de cette recherche, les familles bénéficieront de :

➢ *Pour les familles « intervention »*

- Visites à domicile par une puéricultrice qui se centreront autour de trois temps : un temps d'observation du développement et des progrès du bébé, un temps de jeu avec le bébé et un temps d'écoute des parents.

Rythme des visites à domicile : - 1 tous les mois de la naissance à 1 an
- 1 tous les 2 mois de 1 an à 2 ans
- 1 tous les 6 mois de 2 ans à 4 ans

De 2 ans à 4 ans, la puéricultrice référente contactera la famille par téléphone entre 2 VAD, à savoir à 27 mois, 33 mois, 39 mois et 45 mois. Cet appel permettra à la puéricultrice de s'entretenir avec la famille, de lui rappeler la possibilité d'ajouter des VAD à la demande de la famille ou de lui proposer des VAD supplémentaires si elle-même l'estime nécessaire.
Notre objectif principal étant de répondre aux besoins des familles, l'accompagnement entre 2 et 4 ans se fera en fonction du repérage et de l'expression des besoins émis par les familles, avec au minimum 1 VAD tous les 6 mois et un appel téléphonique 3 mois après la VAD.

Justification de la modification du rythme des VAD entre 2 ans et 4 ans (modification substantielle N°5 au protocole) : suite aux retours cliniques des puéricultrices engagées dans le suivi des familles, nous avons estimé que le suivi imposé était trop intense. Nous avons donc opté pour le libre choix des familles à demander plus de VAD si elles le souhaitent et remplacer 1 VAD sur 2 par un appel téléphonique pour maintenir tout de même le lien avec la famille.

Lors de la VAD réalisée aux 4 ans de l'enfant, un interne en médecine de la PMI accompagnera la puéricultrice référente afin de réaliser un test de développement de l'enfant (échelle GED : Grille d'Evaluation du Développement de l'enfant). Si un retard de développement est constaté, la puéricultrice ou l'interne en médecine proposera un rendez-vous à la famille dans le cadre des consultations de PMI du secteur afin de pouvoir enclencher un suivi ou une orientation si nécessaire.
Le test de développement pourra également être réalisé par l'interne en médecine de la PMI à proximité de la VAD réalisée par la puéricultrice mais au cours d'une VAD distincte, en fonction des contraintes d'agenda de chacun et des souhaits de la famille.
En complément du test de développement de l'enfant, l'interne en médecine de la PMI réalisera un test ERTL4 (Epreuve de Repérage des Troubles du Langage de l'enfant de 4 ans). La version initiale du protocole de l'étude prévoyait déjà de récupérer les données de

l'ERTL4 que les puéricultrices font passer systématiquement dans le cadre des bilans en école maternelle. Afin de respecter les délais de validation de ce test (entre 3 ans 9 mois et 4 ans 6 mois), nous avons jugé préférable qu'il soit réalisé directement au domicile par l'interne en médecine car les dates des bilans faits au sein des écoles sont très variables en fonction des puéricultrices et ne peuvent pas être adaptées à l'âge de chaque enfant de la recherche pour respecter les critères de validité selon l'âge exact.
Dans le cas où la passation du test ERTL4 n'aurait pas lieu lors de la VAD réalisée pour les 4 ans de l'enfant (RDV modifié, RDV annulé, …), les données du test ERTL4 utilisées pour notre étude seront celles récupérées lors du bilan systématique fait en école maternelle comme prévu initialement par le protocole de l'étude.

Justification de la modification de l'ajout d'un test de développement à 4 ans (modification substantielle N°5 au protocole) : il nous a paru important de pouvoir compléter notre évaluation des 4 ans de l'enfant par un test global de développement. Initialement, seul était prévu de récupérer les données de l'ERTL4 qui est fait passé par les puéricultrices dans le cadre des Bilans Ecole Maternelle. Nous n'avions pas la possibilité de faire un test de développement à 4 ans auparavant, n'ayant pas les moyens matériels et humains nécessaires. Désormais, nous pouvons remplir ces critères, c'est pourquoi nous ajoutons cette évaluation, afin d'étoffer le protocole en terme de rigueur scientifique pour répondre à nos objectifs de recherche.

- Visites à domicile réalisées en binôme par la psychologue coordinatrice et la puéricultrice référente de la famille dont l'objectif est l'évaluation de trois domaines : le développement de l'enfant, la parentalité et l'interaction parents-enfants. Ces évaluations peuvent à certains moments être filmées et ces films pourront être restitués aux parents sur simple demande de leur part.

Rythme des visites conjointes à domicile : - 1 à 4 mois
- 1 à 24 mois

Justification de la modification de VAD de 3 mois à VAD de 4 mois (modification substantielle N°3 au protocole) : Après avoir effectué les premières VAD d'évaluation aux 3 mois des bébés, il nous a semblé que les données recueillis au test du BLR n'étaient pas suffisamment représentatives des compétences des bébés, du fait de l'outil. Ces éléments cliniques ont été confirmés par des données de la littérature qui rapportent que « le calcul du quotient de développement ne s'effectue pas avant 4 mois, car, au cours des trois premiers mois ce calcul n'aurait pas de sens car il serait trop élevé » (Manuel du Brunet-Lezine-Revisé 1997).

➢ *Pour les familles « témoins »*

Visites à domicile réalisées par la psychologue coordinatrice et dont l'objectif est l'évaluation de trois domaines : le développement de l'enfant, la parentalité et l'interaction parents-enfants. Ces évaluations peuvent à certains moments être filmées et ces films pourront être restitués aux parents sur simple demande de leur part.

Rythme des visites à domicile : - 1 à 4 mois
- 1 à 24 mois

Visite à domicile réalisée par un interne en médecine de la PMI, afin de réaliser un test de développement de l'enfant (échelle GED : Grille d'Evaluation du Développement de l'enfant). Si un retard de développement est constaté, l'interne en médecine proposera un rendez-vous à la famille dans le cadre des consultations de PMI du secteur afin de pouvoir enclencher un suivi ou une orientation si nécessaire.

Cette visite à domicile par un interne en médecine de la PMI sera réalisée une seule fois, aux 4 ans de l'enfant.

En complément du test de développement de l'enfant, l'interne en médecine de la PMI réalisera un test ERTL4 (Epreuve de Repérage des Troubles du Langage de l'enfant de 4 ans). La version initiale du protocole de l'étude prévoyait déjà de récupérer les données de l'ERTL4 que les puéricultrices font passer systématiquement dans le cadre des bilans en école maternelle. Afin de respecter les délais de validation de ce test (entre 3 ans 9 mois et 4 ans 6 mois), nous avons jugé préférable qu'il soit réalisé directement au domicile par l'interne en médecine car les dates des bilans faits au sein des écoles sont très variables en fonction des puéricultrices et ne peuvent pas être adaptées à l'âge de chaque enfant de la recherche pour respecter les critères de validité selon l'âge exact.
Dans le cas où la passation du test ERTL4 n'aurait pas lieu lors de la VAD réalisée pour les 4 ans de l'enfant (RDV modifié, RDV annulé, …), les données du test ERTL4 utilisées pour notre étude seront celles récupérées lors du bilan systématique fait en école maternelle comme prévu initialement par le protocole de l'étude.

Justification de la modification de l'ajout d'un test de développement à 4 ans (modification substantielle N°5 au protocole) : il nous a paru important de pouvoir compléter notre évaluation des 4 ans de l'enfant par un test global de développement. Initialement, seul était prévu de récupérer les données de l'ERTL4 qui est fait passé par les puéricultrices dans le cadre des Bilans Ecole Maternelle. Nous n'avions pas la possibilité de faire un test de développement à 4 ans auparavant, n'ayant pas les moyens matériels et humains nécessaires. Désormais, nous pouvons remplir ces critères, c'est pourquoi nous ajoutons cette évaluation, afin d'étoffer le protocole en terme de rigueur scientifique pour répondre à nos objectifs de recherche.

- *Pour les familles « témoins » et « intervention »*

  - 10 familles de chaque groupe (intervention et témoin), tirées au sort, bénéficieront d'une visite (à domicile ou à la maternité selon leur convenance), par une psychologue du Centre Psychothérapique de Nancy ayant réalisé la recherche INTERREG (Elise Fidry) afin d'évaluer le développement du bébé et mettre en avant ses compétences auprès des parents. Cette évaluation sera réalisée en aveugle par la psychologue qui ne connaitra pas le groupe auquel la famille appartient (témoin ou intervention).

    Justification de l'ajout du test du Brazelton : Compte tenu des premières VAD d'évaluation réalisées et des données de la littérature (CF justification de la modification de la première VAD d'évaluation), nous avons opté pour une VAD d'évaluation à 4 mois. Cependant, en déplaçant la VAD d'évaluation, une VAD supplémentaire auprès du groupe intervention est réalisée par la puéricultrice et il peut déjà y avoir des effets sur le développement de l'enfant. Il est donc important d'avoir

une première évaluation en amont afin de vérifier l'homogénéité des groupes en terme de développement du bébé, ce qui peut être réalisé par la passation du test de Brazelton, test qui nécessite une formation particulière de l'évaluateur.
Initialement nous n'avions pas proposé ce test faute de professionnel formé et disponible, ce qui est dorénavant possible car une psychologue formée a pu libérer du temps afin de pouvoir réaliser ces 20 Brazelton.

- 10 évaluations GED au moins dans chaque groupe seront filmées afin d'effectuer une double cotation de ce test : une cotation par l'interne de PMI au cours de la VAD et une en aveugle par un des autres internes de PMI formés à l'évaluation GED pour l'étude PERL.
  En effet il est prévu que 3 internes au maximum réalisent le test GED aux 4 ans des enfants, sauf imprévu nécessitant l'intervention d'un interne supplémentaire.
  Les internes réalisant les GED seront tous formés à l'outil avant la passation du test auprès des enfants de la recherche.
  La double cotation (grâce aux entretiens filmés) permettra de s'assurer de la reproductibilité des cotations entre les internes, au sein de chaque groupe (témoin et intervention) et entre les groupes et de pouvoir tenir compte d'un éventuel biais repéré lors des analyses.

2.5 Outils d'évaluation

2.5.1 Données sociodémographiques et familiales

Différentes données socio-démographiques et familiales seront relevées dans les groupes « intervention » et « témoins » (âge, niveau d'étude, activité professionnelle et situation familiale, des deux parents). Nous recueillerons également concernant l'enfant : le sexe, le rang dans la fratrie, le poids de naissance et l'âge gestationnel. Nous relèverons également : le type d'accouchement, la parité, le mode de garde, la scolarisation et l'âge de la première scolarisation, le désir d'enfant, le mode d'alimentation (allaitement ou biberon), et l'investissement du père dans la recherche, présence ou non d'une mesure d'accompagnement de la famille.

Enfin, nous coterons la présence de deux facteurs : le vécu traumatique de la naissance et la pathologie de la carence à partir des critères définis par Lamour en 2015 qui sont au nombre de 4 : carences sanitaires et sociales, absence d'organisation dans la vie quotidienne/discontinuité, histoire des parents marquée par la carence et la maltraitance et fréquence d'une psychopathologie grave chez le parent (mais souvent non reconnue et non traitée).

2.5.2 Evaluation longitudinale

Une évaluation longitudinale sera réalisée dans les deux groupes afin d'établir un profil évolutif des différentes dimensions étudiées :
- le développement du bébé ;
- l'interaction ;
- le vécu de la parentalité ;
- l'investissement/retrait relationnel du bébé ;
- la dépression du post partum ;

- le vécu de l'accompagnement ;
- la symptomatologie psychiatrique maternelle

2.5.3 Evaluation comparative

Les effets de l'accompagnement seront évalués par comparaison à une population témoin, qui n'aura pas bénéficié de l'accompagnement à domicile par les puéricultrices mais uniquement des 3 rencontres d'évaluation à 4 mois, 24 mois et 4 ans.

L'évaluation comparative à 4 et 24 mois chez les groupes intervention et témoins portera sur :
- le développement psychomoteur et langagier de l'enfant;
- le vécu de la parentalité;
- le développement socio-émotionnel de l'enfant;
- l'investissement/retrait relationnel du bébé ;
- la dépression du post partum ;
- la symptomatologie psychiatrique maternelle
- les retards de langage à 4 ans ;
- la nécessité de recours aux soins à 4 ans.

L'évaluation comparative à 4 ans chez les groupes intervention et témoins portera sur l'évaluation globale du développement de l'enfant.

2.5.4 Plan méthodologique

**Age :**	**Naissance**	**4 mois**	**24 mois**	**4 ans**
**POPULATION CONCERNEE :**	10 familles intervention<br>10 familles témoins	INTERVENTION ET<br>TEMOINS	INTERVENTION ET TEMOINS	INTERVENTION ET TEMOINS
Développement de l'enfant	Brazelton	Brunet Lézine Révisé<br>ADBB	Brunet Lézine Révisé<br>ADBB<br>BITSEA	ERTL4<br>GED
Vécu de la parentalité<br>Vécu de l'intervention par les familles		E RP	E RP	
Interactions précoces		PIM13 (analyse des vidéos)	PIM13 (analyse des vidéos)	
Alliance thérapeutique		WAI<br>Uniquement au groupe Intervention	WAI<br>Uniquement au groupeIntervention	
Evaluation des symptômes psychiatriques maternels		SCL-90 R<br>EPDS	SCL-90 R<br>BDI	
Aisance sociale		FAS	FAS	

- Test Brazelton : Echelle d'évaluation du développement du bébé de la naissance à 2 mois. Cet outil évalue les caractéristiques émotionnelles et comportementales du nouveau né à travers un examen clinique associant les parents.
- Test Brunet-Lézine-révisé (BLR) : évaluation du développement psychomoteur de 0 à 30 mois ;
- ERP : Entretien sur les Représentations Parentales ;
- ERTL4 : Epreuve de Repérage des retards de Langage à 4 ans, réalisée de façon systématique par les équipes de Protection Maternelle et Infantile et lors de la VAD des 4 ans de l'enfant ;
- GED : Grille d'Evaluation du Développement de l'enfant de 0 à 6 ans. Cette grille a été validée au Canada et une étude de validation Française est en cours. Cet outil présente l'avantage d'être corrélé avec les résultats obtenus au BLR (test d'évaluation du développement de l'enfant passé à 4 et 24 mois). De plus, ce test associe les parents lors de la passation.
- ADBB : évaluation filmée du retrait relationnel du jeune enfant de 0 à 24 mois (vidéo);
- EPDS : Edinburgh Postnatal Depression Scale. Echelle d'évaluation de la dépression du post partum ;
- BDI : Inventaire de Dépression de Beck. Echelle qui évalue la sévérité de la dépression chez l'adulte ;
- BITSEA: Brief Infant-toddler Social and Emotional Assement. Echelle de dépistage précoce des troubles du comportement et des retards d'acquisition entre 1 et trois ans.
- PIM 13 : Parent-infant, early Interactions Measure 13 items : outil élaboré par Mme Sophie BUCHHEIT et le Pr Fabienne LIGIER. Cette grille intègre des items permettant d'évaluer la qualité des interactions précoces et la sensibilité maternelle. L'attachement est en trame de fond de cette grille.
- FAS : Echelle d'évaluation de l'aisance sociale afin de déterminer le niveau socio-économique des familles
- SCL-90 R : Symptome Check List : questionnaire global d'autoévaluation des symptômes psychiatriques
- WAI : Working Alliance Inventory (WAI) (Horvath, 1994) [27]: il s'agit d'une échelle d'alliance thérapeutique largement utilisée dans les études anglo-saxonnes, qui a été traduite en français et adaptée au contexte des consultations précoces parents-jeune enfant. L'échelle comprend 12 questions explorant quatre dimensions de l'alliance: les liens thérapeute-parent (3 questions), l'alliance thérapeute-enfant (3 questions), la dimension "positive" des objectifs et des tâches (3questions) et la dimension "négative" des objectifs et des tâches (3 questions dont la réponse est inversée par rapport aux autres). Les réponses se font sur une échelle en 7 points dont 3 sont définis (1= pas du tout d'accord; 4=modérément d'accort, 7=tout à fait d'accord). Dans cette étude, les échelles seront légèrement modifiées (remplacement de "consultations" par "rencontres", et de "consultant" par "puéricultrice") afin de s'adapter au contexte du travail de PMI. La puéricultrice qui suivra la famille et la mère rempliront les échelles d'évaluation de l'alliance.

La majorité des tests utilisés ont été validés en français, la GED l'a été au Canada et une étude de validation Française est en cours et ils présentent de bonnes qualités psychométriques. Seuls l'Entretien sur les Représentations Parentales et l'échelle PIM 13 : Parent-infant, early Interactions Measure 13 items n'ont pas été scientifiquement validées. Cependant, l'ERP est un outil qui a été spécifiquement conçu pour la première recherche-action (INTERREG). Pour élaborer cet instrument, nous nous sommes basés sur les apports de Stern et al. (1989) à propos

de l'entretien « R » et du PDI qui sont des méthodes d'évaluation des représentations maternelles. La PIM 13 quant à elle a été construite par des membres de notre équipe scientifique et médicale (Mme Sophie BUCHHEIT, Pr Fabienne LIGIER). Nous avons intégré des items permettant d'évaluer la sensibilité maternelle et la qualité des interactions précoces. L'attachement étant en trame de fond de cette grille. Pour la construction de cette grille d'observation nous nous sommes inspirés de plusieurs tests validés (Français et/ou internationaux) : CIB (Coding Interactive Behavior), CARE-INDEX, MBQS (Maternal Behavior Q-Sort), AQS (Attachment Q-Sort), DMC (Dyadic Mutuality Code), GRS (Global Rating Scale), GEDAN (Grille d'Evaluation du Dialogue Adulte-Nourrisson). Nous avons également consulté le guide de Steinhauer, l'ADBB (Alarme Détresse-BéBé) et le PIPE (Pédiatric Infant Parent Exam). La création de cet outil nous a été recommandée par le Professeur Antoine Guedeney, professeur de psychiatrie spécialisé dans notre domaine de recherche.

2.5.5 Analyse des processus et mécanismes

A noter qu'à l'évaluation longitudinale et comparative de cette recherche s'associe une analyse des processus et mécanismes qui sera réalisée par l'équipe du laboratoire APEMAC (Ecole de santé publique, laboratoire de Maladies chroniques, santé perçue et processus d'adaptation. Approches épidémiologiques et psychologiques). Il s'agira plus spécifiquement :

1) de définir et valider la théorie d'intervention (qu'est-ce que produisent les leviers mobilisés et comment, quels sont les mécanismes reliant l'intervention à ses résultats),
2) de décrire l'implantation et les processus en jeu,
3) d'analyser l'effet du contexte, notamment social, sur les processus et les résultats.

Cette analyse fera l'objet d'un protocole spécifique de l'Ecole de Santé Publique de Nancy.

<u>2.6 Mode de traitement des données</u>

2.6.1 Analyse descriptive

Une analyse descriptive sera faite pour :
- les caractéristiques socio démographiques
- les facteurs de vulnérabilités

2.6.2 Analyse statistique

Une analyse statistique et qualitative intermédiaire sera effectuée deux ans après le début de l'étude afin de confirmer qu'il n'y a pas d'éléments délétères pour les participants. Cette analyse statistique sera effectuée par la coordinatrice de la recherche ainsi que par Madame la Professeure Fabienne LIGIER (Docteure en santé publique).

Les tests statistiques utilisés seront les suivants :

- Le test du t de student sera utilisé pour comparer les variables quantitatives :
  * comparer l'homogénéité des deux groupes (intervention et témoins) concernant les variables âge des parents, poids de naissance de l'enfant, âge gestationnel.
  * comparer les résultats obtenus au Brunet-Lezine, à l'ERTL4, à la BDI, la BITSEA, et à l'ADBB entre les deux groupes.
  * Analyser l'impact de la tonalité des représentations parentales sur les scores obtenus aux différentes échelles.

- Le test du Khi 2 (χ^2) sera utilisé pour :
  * comparer l'homogénéité des deux groupes pour les variables qualitatives concernant le sexe de l'enfant, le rang dans la fratrie, le niveau d'étude des parents, l'activité professionnelle des parents et la structure familiale Pour les familles concernées, il y aura également une comparaison des scores au test de Brazelton.
  * observer si une différence apparaît dans la tonalité des Représentations Parentales à l'entretien (vécu de la parentalité) entre les deux groupes.

- Le coefficient de corrélation Rho de Spearman sera utilisé pour les corrélations entre les scores obtenus aux différentes échelles (Brunet-Lézine, ADBB, EPDS, BDI, BITSEA, WAI, FAS, SCL-90 R).
L'ensemble des conditions d'utilisation des tests seront vérifiées avant leur utilisation.

3. PRECAUTIONS METHODOLOGIQUES ET GARANTIES ETHIQUES

3.1 Précautions méthodologiques

3.1.1 Les tests

L'ensemble des outils méthodologiques répondent à différentes caractéristiques :
- ils doivent rendre compte de données comparatives et transmissibles ;
- ils ne doivent pas être trop intrusifs, la passation ne doit pas être trop lourde, ni pour l'intervenant, ni pour le parent (pour ne pas "aller contre" l'objectif de prévention, et pour que le programme soit reproductible facilement) ;
- les grilles d'observation doivent être les plus descriptives possibles, pour enrichir la qualité des données et bien différencier le temps d'évaluation et le temps d'analyse ;
- les différents outils vont dans le même sens et participent à nos objectifs de prévention (on cherche à mettre en valeur les ressources, les compétences, à favoriser l'interaction…).
- les grilles et questionnaires utilisés sont validés en français (ou en anglais) et pour ceux ne l'étant pas, ils ont fait l'objet de publications françaises et/ou internationales, à l'exception de la grille PIM13, néanmoins celle-ci a été construite en s'appuyant sur des tests validées (cf point 2.5.4) .

3.1.2 Anonymat

Lorsque les familles seront considérées comme faisant partie de l'étude nous procéderons à l'anonymisation. Pour ce faire, un chiffre sera attribué à l'inclusion de chaque famille : 1 pour intervention 1 et T1 pour Témoin 1. Les données nominatives ne seront pas conservées au même endroit et sur le même support que les données anonymisées et la liste de numéros attribués, afin de garantir l'anonymat et qu'aucun regroupement ne puisse se faire.

3.1.3 Recueil des données

L'ensemble des données récoltées durant les 4 années de recherche seront recueillies sur une fiche d'observation papier et constitueront les documents sources associés aux résultats des évaluations. Les données nécessaires à la recherche seront consignées dans un ordinateur portable et un disque dur externe (de façon anonymisé) ; seule Madame BUCHHEIT y aura accès. L'ordinateur ne sera pas accessible à un tiers, verrouillé par un mot de passe et une carte (Carte Professionnelle Sécurisée) et sera consigné dans une armoire, sous clé, au Centre Médico

Psychologique pour enfants et adolescents de Vandoeuvre, résidence administrative de Madame Buchheit.
Le code attribué lors de l'inclusion de la famille sera le seul à figurer sur les données recueillies.

Les règlementations de la CNIL seront appliquées pour l'identification des participants. Nous ne recueillerons pas de dates de naissances, néanmoins, si elle devait être mentionnée, elle se limiterait aux mois et année de naissance (le jour étant trop identifiant).

L'ensemble des données recueillies seront conservées et détruites 15 ans après l'arrêt de la recherche.

3.2 Garanties éthiques

3.2.1 Comité scientifique

Cette recherche est soutenue par un comité scientifique pluridisciplinaire qui se réunit tous les deux mois afin de faire le point sur l'évolution de la recherche et s'assurer que tous les critères éthiques et méthodologiques sont respectés.
Ce groupe de travail est constitué de :

- S. BUCHHEIT, psychologue clinicienne, coordinatrice du projet, Centre Psychothérapique de Nancy
- Pr. B. KABUTH, Professeur de pédopsychiatrie, Université de Lorraine, Chef de Pôle de Pédopsychiatrie du Centre Psychothérapique de Nancy
- Pr F. LIGIER, pédopsychiatre, docteure en Santé Publique, Centre Psychothérapique de Nancy.

Ainsi qu'un ou plusieurs représentants des structures suivantes : ARS Grand Est, Conseil Départemental, PMI, CAF du Lunévillois, Département prévention et promotion en santé mentale du Centre Psychothérapique de Nancy ; ainsi que des pédopsychiatres, psychologues et cadres de santé du Centre Psychothérapique de Nancy et des cadres de santé du Conseil Départemental.

Des représentants de l'Ecole de Santé Publique de Nancy apporteront un avis méthodologique concernant le projet dans son ensemble et seront présents lors des réunions du Comité Scientifique pendant toute la durée de l'évaluation des processus et mécanismes.

3.2.2 Documents

Consentement
Après que les familles aient donné leur accord pour participer à cette recherche, ils devront signer le formulaire de consentement. Un exemplaire de ce consentement leur sera remis ainsi qu'un exemplaire de la note d'information.
Le formulaire de consentement devra être signé par les deux parents ayant l'autorité parentale, et à défaut, par le parent ayant l'autorité parentale exclusive dans le cas ou le père n'a pas reconnu l'enfant ou si ce dernier est absent lors de la présentation de l'étude (comme le prévoit l'article L1122-2 du Code de la Santé Publique *« l'autre titulaire de l'exercice de l'autorité parentale ne peut donner son autorisation dans des délais compatibles avec les exigences méthodologiques propres à la réalisation de la recherche, au regard de ses finalités »).*
Néanmoins la coordinatrice de la recherche reste disponible pour toute demande d'information complémentaire de la part du père.

Note d'information
La note d'information sera lue avec les familles et expliquée avant qu'un exemplaire leur soit fourni.

Droit d'accès à l'image
Un document de droit d'accès à l'image devra également être signé par les parents pour recueillir leur consentement et nous autoriser à utiliser les vidéos que nous réaliserons pour la cotation des évaluations, telles que décrites dans le protocole. Un exemplaire leur sera également remis.
Ce document devra, tout comme le consentement éclairé, être signé par les deux parents ayant l'autorité parentale sur l'enfant.

Protection des données :

- CNIL

Cette étude n'entre pas dans le champ de la méthodologie de référence MR001 de la CNIL du fait de la réalisation de vidéos identifiantes auprès des familles recrutées pour l'étude.
Une autorisation de la CNIL est requise avant mise en œuvre de l'étude. Les démarches nécessaires ont été effectuées par le promoteur de cette étude.

- RGPD

Conformément au Règlement Général sur la Protection des Données (RGPD), les personnes participant à cette recherche seront informées, via la notice d'information et le formulaire de consentement, des droits suivants :

- de l'identité et des coordonnées du responsable du traitement des données, à savoir le promoteur
- des coordonnées du délégué à la protection des données
- du fondement juridique sur lequel repose le traitement des données
- de la nature et de la finalité des données recueillies dans le cadre de la recherche ainsi que du délai de conservation de ces données
- de la possibilité d'arrêter l'étude à tout moment et de la conservation, par le promoteur, des informations recueillies (sauf indication contraire de la personne concernée).
- de leurs droits d'accès, de rectification, d'opposition, de limitation et d'effacement des données recueillies dans le cadre de la recherche. Ces droits pourront s'exercer à tout moment de la recherche en effectuant une demande auprès du Délégué à la Protection des Données du promoteur
- de la possibilité, en cas de problème/désaccord, d'effectuer une réclamation auprès de la CNIL.

Le promoteur s'engage à répondre à toute demande d'accès aux données dans un délai d'1 mois maximum. Ce délai pourra être prolongé de 2 mois en fonction de la complexité et du nombre de demandes. Par ailleurs, seul le personnel habilité par le promoteur (investigateurs, ARC, TEC) et les représentants des autorités de santé pourront avoir accès à ces informations.

3.2.3 Vigilance

Cette étude fait partie des Recherches Impliquant la Personne Humaine, de catégorie 2 (risques et contraintes minimes) selon la loi N°2012-300 du 5 mars 2012 (loi Jardé).

A ce titre, aucun circuit spécifique de suivi de la vigilance n'est requis pour cette étude de la part du promoteur.

Seuls les effets indésirables constatés par l'investigateur ou signalés par les familles pendant leur participation à l'étude seront consignés dans le cahier d'observation de l'étude.

Si ces effets indésirables relèvent d'une vigilance sanitaire (pharmacovigilance pour les effets indésirables médicamenteux, matériovigilance pour un effet lié à un dispositif médical,…), ils seront transmis par l'investigateur selon la procédure habituelle de déclaration à la vigilance sanitaire concernée, sans avertir le promoteur.

4. BENEFICES/RISQUES DE LA RECHERCHE

4.1 Bénéfices

Nous nous attendons à mesurer un effet bénéfique d'une intervention conjointe puéricultrice (de la Protection Maternelle Infantile) et psychologue (de pédopsychiatrie) sur le développement de l'enfant et le vécu de la parentalité.
Nous attendons une diminution importante des retards de développement et des retards de langage et un meilleur vécu de la parentalité pour les familles bénéficiant de l'accompagnement.

4.2 Risques

Il n'y a pas de risque identifié dans cette recherche-action.
Il est important de spécifier que les cohortes « témoins » et « intervention » pourront bénéficier d'un suivi PMI et CMP classique si elles le souhaitent.
Cette étude ne pénalise en rien la cohorte « témoins » dans les suivis habituels qui auraient pu et qui peuvent se mettre en place dans la période périnatale.
De plus, dans l'éventualité où des difficultés seraient détectées lors des visites d'évaluation de la population témoin, nous informerons les parents de nos inquiétudes et ils seront orientés vers une structure de soins adaptée aux difficultés relevées. Cette configuration n'entravera pas leur participation à cette recherche.

4.3 Indemnités

Il n'y a pas de dédommagement financier prévu pour cette recherche. Cette recherche n'entraîne aucun coût financier pour les familles y participant.

Il n'y a pas d'interdiction de participer simultanément à une autre recherche pendant la participation à la recherche-action PERL.
Il n'y a pas de période d'exclusion durant laquelle la participation à une autre recherche est interdite.

5. FINANCEMENTS DE LA RECHERCHE

5.1 Financement des postes

Cette recherche a obtenu un financement pluri-institutionnel :

- le conseil départemental met à disposition les puéricultrices de secteur pour participer à la recherche (1.60 ETP) ;
- le Centre Psychothérapique de Nancy finance le poste de la psychologue qui coordonne et évalue la recherche ainsi que le poste de la psychologue qui fera les supervisions (1 ETP) ;
- l'Agence Régionale de la Santé apporte un soutien financier au poste de psychologue ;
- la Caisse d'Allocations Familiale apporte un soutien financier au poste de psychologue.

5.2 Budget

La totalité du budget pour financer la recherche-action PERL a été accordé. Ce budget comprend :
- les 10 mallettes de jeux dont disposeront les puéricultrices pour faire les visites à domicile ;
- l'ensemble des tests utilisés par la psychologue lors des évaluations ;
- l'ordinateur et le disque dur externe permettant de traiter les données recueillies ;
- le remboursement des frais de transports de la psychologue ;
- l'assurance.

6. DONNEES RECUEILLIES ET DOCUMENTS SOURCES

Description des variables recueillies
Les données de l'étude seront recueillies selon les modalités décrites au paragraphe 3.1.3.
Les informations recueillies dans le cadre de cette étude sont décrites au paragraphe 2.5.

Identification des données sources
Les documents sources seront constitués par l'ensemble des informations et résultats aux tests utilisés dans le cadre de cette étude, figurant dans un dossier spécifique à la recherche et centralisé au CMP enfants et adolescents de Vandoeuvre.
Les informations suivantes figureront dans le dossier de chaque famille participant à l'étude :
- titre de l'étude,
- date d'information et d'inclusion du patient dans l'étude (signature du consentement),
- les différentes visites du patient dans le cadre du protocole,
- la survenue d'Evénements Indésirables (Graves).

L'investigateur coordonnateur s'engage à donner un accès direct à l'ensemble de ces documents aux personnes mandatées par le promoteur, ainsi qu'aux représentants des autorités de santé.

7. CONTRÔLE ET ASSURANCE QUALITE

Le contrôle qualité sera réalisé par un Attaché de Recherche Clinique (ARC) mandaté par le promoteur en fonction du niveau de monitoring défini au préalable.

8. CONSIDERATIONS ETHIQUES ET REGLEMENTAIRES

Il s'agit d'une étude entrant dans le cadre de la loi 2012-300 (dite loi Jardé) du 5 mars 2012, catégorie 2.

Respect des dispositions réglementaires en vigueur
La recherche sera conduite conformément au protocole, aux bonnes pratiques et aux dispositions législatives et réglementaires en vigueur
Le protocole est en conformité avec les principes éthiques établis par la déclaration d'Helsinki

Protection des personnes
Avant de débuter les inclusions, le protocole sera soumis à l'avis du Comité de Protection des Personnes Nord-Ouest IV à Lille.
Il sera transmis pour information à l'ANSM.
Les modalités de recueil du consentement sont décrites au paragraphe 3.2.2

Assurance
Le promoteur de l'étude a souscrit une assurance garantissant sa responsabilité civile et celle de tout intervenant, indépendamment de la nature des liens existant entre les intervenants et le promoteur.

Inscription au fichier national VRB (Volontaires Recherches BioMédicales)
Il n'y a pas d'interdiction de participer simultanément à une autre recherche pendant la participation à cette étude.
Il n'y a pas de période d'exclusion durant laquelle la participation à une autre recherche est interdite.
Aucune indemnisation des participants n'est requise dans le cadre de cette étude.
De ce fait, cette étude ne nécessite pas l'inscription des participants au fichier VRB.
