# Supplementary material for: Development and validation of the MIPPE: A novel dyadic assessment tool for early parent-child interactions in clinical practice
Source: PLoS One. 2026 Apr 24;21(4):e0347521. doi: 10.1371/journal.pone.0347521 (PMC13108784; doi:10.1371/journal.pone.0347521)
Supplement: Attachment — Submitted filename: Response_to_Reviewers.docx [file pone.0347521.s010.docx]

Emmanuelle DUFAIT

University of Lorraine, France

BioSE Doctoral School

Emmanuelle.dufait@gmail.com

Nancy, March 27, 2026

**Subject: Response to Reviewers.**

Manuscript ID: PONE-D-25-61400

Title: Development and validation of the MIPPE: a novel dyadic assessment tool for early parent-child interactions in clinical practice

Journal: PLOS ONE

Dear Dr. Waechter and Editorial Team,

We sincerely thank you for the careful evaluation of our manuscript and for the opportunity to revise and resubmit our work. We are grateful to the Academic Editor and to Dr. Barbara Landon for their thorough and constructive feedback, which has led to meaningful improvements in the manuscript.

We have addressed each comment point by point below. All changes are visible in the Revised Manuscript with Track Changes. A clean version without tracked changes is also provided.

We remain available should any further clarification be needed.

Sincerely,

Emmanuelle Dufait, on behalf of all co-authors

# Journal Requirements

## Requirement 1 — PLOS ONE style and file naming

We have carefully reviewed the PLOS ONE formatting guidelines and have ensured that the revised manuscript and all associated files comply with the required style, including file naming conventions for Supporting Information files (S1File through S12 File).

## Requirements 2 & 3 — Data availability statement

Additionally, in accordance with PLOS ONE recommendations, the full validation protocol has been deposited on protocols.io (DOI: https://dx.doi.org/10.17504/protocols.io.8epv5y59dl1b/v1). A private access link for reviewers is available at: https://www.protocols.io/private/31E95DEA29F811F185570A58A9FEAC02 (to be removed before publication).

The underlying data supporting the findings of this manuscript consist of MIPPE item scores and total scores for the 143 professional raters across 6 videos, along with basic sociodemographic variables (child age, child sex, maternal education level, professional situation). These data are provided as a Supporting Information file (S12 File).

Three additional data sources used in this study cannot be shared publicly for the following reasons: (1) The PERL video recordings. The 228 video recordings of parent-child dyadic interactions were filmed during home visits as part of the PERL longitudinal study. These videos contain identifiable images of minor children and their families, collected under an ethics protocol (CPP Nord-Ouest IV, IDRCB: 2017-A00896-47) and CNIL authorization (Decision DR-2018-10) that do not permit unrestricted public deposition. The consent forms signed by participants explicitly authorize data use for research, evaluation, educational purposes, and scientific presentations, but do not authorize open online sharing of identifiable audiovisual material. (2) The Revised Brunet-Lézine and ADBB data. These data were collected within the broader PERL longitudinal study and are the subject of ongoing publications by the PERL research group. Their public deposition at this stage would conflict with current publication embargoes and with the data governance agreement between the research team and the institutional sponsor (Centre Psychothérapique de Nancy, Laxou, France). We note in particular that the convergent validity analyses relied on two distinct Brunet-Lézine measures: the language subscale (which showed significant correlations with MIPPE scores at 4 and 24 months) and the total developmental score (for which no significant correlation was found at 4 months, consistent with the limited differentiation of global developmental indices at this early age). This distinction is now explicitly stated in the manuscript. (3) Sensitive psychosocial variables. The PERL dataset also includes sensitive family-level variables (including substance addiction indicators) collected as part of the preventive intervention protocol. Given the small sample size (N = 123 dyads) and the sensitive nature of this information, sharing these variables publicly would risk indirect identification of participants despite pseudonymization.

We therefore request a partial data sharing exemption for the Brunet-Lézine and ADBB datasets, in accordance with PLOS ONE's data policy provision allowing restrictions where public deposition would conflict with the protocol approved by the research ethics board or with ongoing publication commitments. The MIPPE validation data (scores and sociodemographic variables) are fully available and have been deposited in S12 File.

## Requirement 4 — Full ethics statement in the Methods section

The manuscript already contained a detailed ethics section. We have further clarified the informed consent procedure for professional raters, specifying that they provided implicit informed consent through their voluntary access to a dedicated, link-restricted online rating platform. As data collected from raters were fully anonymous and did not allow for individual identification, no written consent form was required. The platform has since been taken offline to ensure data protection. The revised statement now explicitly mentions whether consent was written or verbal for each participant group, in accordance with PLOS ONE requirements.

## Requirement 5 — Captions for supporting information files

Captions for all Supporting Information files (S1–S12) have been added at the end of the manuscript, in accordance with PLOS ONE Supporting Information guidelines. In-text citations have been updated accordingly (notably, the MIPPE scale is now cited as "S1 File" throughout the manuscript).

## Requirement 6 — Corrupted supporting information file

The file previously identified as corrupted ("consentement0510.docx and renamed_a3a62.pdf") has been removed. All Supporting Information files have been reorganized, properly named, and re-uploaded in PDF format. Each file has been verified for accessibility prior to submission.

## Requirement 7 — Previously published works recommended by reviewer

Reviewer #1 did not recommend specific previously published works for citation. No action was required on this point.

## Requirement 8 — Reference list verification

The reference list has been thoroughly revised. All references now include Digital Object Identifiers (DOIs) in Vancouver format. Reference [2] (Bozicevic et al.), which had been cited prior to publication, has been updated with the full published citation (J Reprod Infant Psychol. 2025;43(5):1301–1336). Reference [5] (Fiori-Cowley et al., 1996) remains a grey literature publication for which no DOI is available; this has been noted. No retracted articles were identified in the reference list. The numbering of all in-text citations has been updated to reflect Vancouver sequential order (order of first appearance in the text). In addition, the two references previously cited in support of the COSMIN criteria ([1] Bayoğlu 2013 and [17] Shanley & Niec 2011) were not COSMIN-specific references and have been replaced by the two foundational COSMIN publications: Mokkink et al. (2010, J Clin Epidemiol) for the taxonomy and terminology [18], and Mokkink et al. (2010, Qual Life Res) for the methodological checklist [19].

# Reviewer #1 — Dr. Barbara Landon

We sincerely thank Dr. Landon for her positive overall assessment and her specific, constructive comments. Her evaluation was generous and her remarks highly relevant. We address each comment below.

## Comment 1 — "Identify KMO in the first instance."

***Reviewer's comment:*** *Identify KMO in the first instance.*

**Response:** We thank Dr. Landon for this observation. The Kaiser-Meyer-Olkin (KMO) index was indeed introduced without its full designation at first mention. In the revised manuscript, the term "Kaiser-Meyer-Olkin (KMO) index" now appears in full at its first occurrence in the Methods section (MIPPE scale validation process), with the abbreviation used thereafter. The redundant definition that appeared later in the Results section has been removed.

## Comment 2 — Context of the measure and the protocol

***Reviewer's comment:*** *Consider providing a bit more context, i.e. how the measure aligns with the protocol, how the protocol came about.*

**Response:** We thank Dr. Landon for this valuable suggestion. The Scale Development section has been substantially expanded to provide the requested context. The revised section now describes: the origins of the PERL program — a single-center randomized controlled trial launched in 2017 in Meurthe-et-Moselle (Grand-Est, France), coordinating maternal and child welfare services (PMI) and child psychiatry to evaluate the effects of preventive home visiting on child development and parental well-being; the reason why existing validated scales could not be used within the PERL context (extensive training requirements, long administration times, lack of French translation); and how the MIPPE was developed as a direct response to this gap, on the advice of Professor Antoine Guédeney, a recognized expert in perinatal prevention and attachment, with iterative refinement between 2020 and 2024 using the PERL video corpus.

## Comment 3 — "Explain 'professional situation' in Table 1."

***Reviewer's comment:*** *Explain "professional situation" in Table 1.*

**Response:** The term "professional situation" refers to the mother's employment status at the time of inclusion in the PERL study. This concept reflects a French administrative category (statut professionnel) that does not have a direct equivalent in English. A footnote (^a^) has been added beneath Table 1 clarifying that "professional situation" refers to the mother's employment status at the time of inclusion, and that "Indefinite Contract" corresponds to a permanent open-ended employment contract (CDI, Contrat à Durée Indéterminée), the standard form of long-term employment in France.

## Comment 4 — "Explain Items 8 and 9 in Table 2."

***Reviewer's comment:*** *Explain Items 8 and 9 in Table 2.*

**Response:** Items 8 and 9, which assess the evaluator's subjective experience when observing the child and the parent respectively, were not sufficiently explained in the original manuscript. We chose not to add a footnote directly beneath Table 2, as the table already presents the response distribution for all 9 items and adding a note specifically for Items 8 and 9 would have disrupted its readability. Instead, we have made two complementary additions at the text level: (1) a paragraph preceding Table 2 now introduces all 9 MIPPE items and explicitly describes Items 8 and 9 as capturing the clinician's emotional response during observation — a countertransferential dimension that serves as a clinical indicator of interactional quality; (2) the corresponding passage in the Discussion section has been enriched to explain that this subjective dimension, rarely included in existing scales, can help identify which member of the dyad is experiencing the most difficulty in the interaction. We believe that this placement allows for a more thorough conceptual explanation than a table footnote would have permitted.

## Comment 5 — Table 6

***Reviewer's comment:*** *Table 6 is impressive and adds to your scale's credibility significantly.*

**Response:** We sincerely thank Dr. Landon for this encouraging comment. Table 6, which presents the distribution of judges across socio-professional categories and demonstrates the scale's discriminant validity across diverse professional backgrounds, reflects the considerable effort invested in assembling a representative and heterogeneous sample of early childhood professionals. We are pleased that this aspect of the validation was recognized as a strength of the study.

## Comment 6 — Missing Appendix 1 (the MIPPE scale)

***Reviewer's comment:*** *Although there are numerous supporting documents I did not find Appendix 1, the scale itself, which may help to explain items 8 and 9, and perhaps also the parental intrusiveness item. Please verify that the Appendix is attached.*

**Response:** We sincerely apologize for this oversight in the original submission. The MIPPE scale had been uploaded under the category "Other" rather than "Supporting Information," which prevented Dr. Landon from locating it. The complete MIPPE scale (9 items with scoring instructions) is now provided as S1 File, correctly classified as Supporting Information. It is cited in the manuscript text at two points: in the Introduction ("(MIPPE) (S1 File), a new assessment scale...") and in the Scale Development section ("The final version of the MIPPE scale is provided in S1 File"). We hope that access to the full scale will clarify both the scoring of Items 8 and 9 and the parental intrusiveness item (Item 7), as noted by Dr. Landon.

We hope that these revisions adequately address all the points raised and that the manuscript is now suitable for publication in PLOS ONE. We remain at your disposal for any further information.

Sincerely,

Emmanuelle Dufait

Bernard KABUTH, Fabienne LIGIER, Sophie BUCHHEIT, Alexis RENARD
Pôle du Grand Nancy, Centre Psychothérapique de Nancy (Nancy Psychotherapeutic Center), Laxou, France
[Bernard.KABUTH@cpn-laxou.com](mailto:Bernard.KABUTH@cpn-laxou.com); [Fabienne.LIGIER@cpn-laxou.com](mailto:Fabienne.LIGIER@cpn-laxou.com); [Sophie.BUCHHEIT@cpn-laxou.com](mailto:Sophie.BUCHHEIT@cpn-laxou.com); alexis.renard3@etu.univ-lorraine.fr
